# Efficacy and Safety of Subcutaneous and Oral Semaglutide Administration in Patients With Type 2 Diabetes: A Meta-Analysis

**DOI:** 10.3389/fphar.2021.695182

**Published:** 2021-10-06

**Authors:** Ping Zhong, Hai Zeng, Miaochun Huang, Guoxin He, Zhixia Chen

**Affiliations:** ^1^ Department of Acupuncture and Moxibustion, The Second Clinical College of Guangzhou University of Chinese Medicine, Guangzhou, China; ^2^ College of Traditional Chinese Medicine, Jinan University, Guangzhou, China; ^3^ Emergency Department, Guangdong Provincial Hospital of Chinese Medicine, Guangzhou, China; ^4^ Department of Spleen and Stomach Diseases, Shenzhen Hospital of Beijing University of Chinese Medicine, Shenzhen, China; ^5^ Guangdong Provincial Hospital of Chinese Medicine, Guangzhou, China

**Keywords:** semaglutide, type 2 diabetes, meta-analysis, randomized controlled trials, treatment outcomes

## Abstract

**Background:** This meta-analysis aimed to combine the data available from clinical trials to assess the effects of subcutaneous and oral semaglutide administration on glycemic control, weight management, and safety outcomes in patients with type 2 diabetes (T2D).

**Methods:** We systematically searched for phase 3 randomized controlled trials (RCTs) that compared semaglutide with placebo or other anti-diabetic drugs in T2D patients. The primary outcome was the change from baseline in glycated hemoglobin (HbA_1c_) levels. Secondary efficacy endpoints included the change from baseline in body weight, achievement of HbA_1c_ targets, and clinically significant weight loss. Key safety outcomes were also assessed.

**Results:** In this meta-analysis, 24 trials with a total of 22185 patients were included. Subcutaneous semaglutide administration reduced HbA_1c_ levels (weighted mean difference [WMD]: −1.14% and −1.37%, for 0.5 mg and 1 mg, respectively) and body weight (WMD: −2.73 kg and −4.09 kg, for 0.5 mg and 1 mg, respectively) when compared with placebo; its efficacy was also superior to other anti-diabetic drugs in reducing HbA_1c_ levels (WMD: −0.71% and −0.86%, for 0.5 mg and 1 mg, respectively) and body weight (WMD: −2.65 kg and −3.78 kg, for 0.5 mg and 1 mg, respectively). Oral semaglutide administration was superior to placebo in decreasing HbA_1c_ levels (WMD: −0.96% and −1.02%, for 7 mg and 14 mg, respectively). Moreover, oral administration of 14 mg of semaglutide also showed a significant reduction in HbA_1c_ levels (WMD: −0.36%) compared with other anti-diabetic drugs. Furthermore, oral semaglutide administration resulted in substantial weight loss compared with other anti-diabetic drugs (WMD: −1.53 kg and −1.73 kg, for 7 mg and 14 mg, respectively). Notably, subcutaneous and oral semaglutide administration also resulted in higher numbers of patients achieving the targets of HbA_1c_ levels and weight loss than placebo and other anti-diabetic drugs. Overall, we noted no clear evidence of detrimental effects on safety endpoints due to semaglutide treatment, except for some gastrointestinal adverse events.

**Conclusion:** Both subcutaneous and oral semaglutide administration could enable the achievement of sufficient glycemic control and weight management without increasing the risk of hypoglycemia, which were effective and safe for the treatment of T2D.

## Introduction

Globally, type 2 diabetes (T2D) is a major public health issue; its prevalence was estimated to reach a remarkable proportion in 2017, affecting 463 million people worldwide (James. et al., 2018). The pathogenesis of T2D involves insulin secretory defects and insulin resistance that clinically manifests as hyperglycemia (Skyler et al., 2017; Riddle. et al., 2019a). Patients with T2D are at high risk of developing a range of complications, including cardiovascular events, chronic kidney disease, diabetic retinopathy, and neuropathy (Das et al., 2018; Davies et al., 2018; Riddle. et al., 2019f). In addition, high fasting plasma glucose also increases the risk of disability and death (Stanaway. et al., 2018).

Glucagon-like peptide-1 (GLP-1) receptor agonists (GLP-1 RAs) are efficacious pharmacological therapies for T2D, which lead to a reduction in plasma glucose levels and induce weight loss without increasing the associated risk of hypoglycemia (Nauck, 2016). Importantly, GLP-1 RAs appear to reduce the risk of major adverse cardiovascular events, microvascular complications, and mortality in patients with T2D (Dicembrini et al., 2017; Bethel et al., 2018; Giugliano et al., 2019; Zelniker et al., 2019). To date, almost all GLP-1 RAs are available only as injectable drugs (Riddle. et al., 2019d). Semaglutide is a novel GLP-1 RA that can be administered weekly by subcutaneous injection. More recently, an oral formulation of semaglutide has been approved by the U.S. Food and Drug Administration (FDA), which has expanded the available treatment options for T2D patients (Meier, 2021).

Both subcutaneous and oral semaglutide administration have already been investigated in several phase 3 randomized controlled trials (RCTs). Individual trials have proven that they can effectively lead to good glycemic control and clinically significant weight loss in patients with T2D (Sorli et al., 2017; Aroda et al., 2019). In addition, their cardiovascular risk profiles are comparable with placebo (Marso et al., 2016; Husain et al., 2019). However, the overall estimated effects and safety of subcutaneous and oral semaglutide administration for treating T2D that combine the data available from the most recent phase 3 RCTs are still lacking. Therefore, we aimed to perform a meta-analysis to examine their treatment outcomes in T2D.

## Methods

This meta-analysis (protocol registration No. CRD42019147387) was conducted in compliance with the Preferred Reporting Items for Systematic Reviews and Meta-Analyses (PRISMA) guidelines (Supplementary Material) (Moher et al., 2009).

### Search Strategy

We obtained the eligible RCTs from PubMed, Embase, and the Cochrane Library. All RCTs that compared subcutaneous or oral semaglutide administration with placebo or other anti-diabetic drugs in T2D patients were retrieved from these electronic databases. Detailed search strategies are provided in the Supplementary Material. The literature search included studies until March 12, 2021. No language restrictions were imposed. The reference lists of relevant reviews, meta-analyses, and included studies were manually scrutinized to seek additional eligible studies.

### Study Selection

The criteria for inclusion were as follows: 1) phase 3 RCTs that enrolled patients (18 years of age or older) with T2D and compared subcutaneous or oral semaglutide administration with placebo or other anti-diabetic drugs; 2) reporting relevant treatment outcomes and with a minimum intervention duration of 8 weeks; 3) the percentage of patients completing treatment in clinical trials exceeded 70%. Phase 1 and phase 2 clinical trials, observational studies, and real-world studies were all excluded. All titles and abstracts of the retrieved articles were screened by two independent authors. After eliminating the irrelevant studies, the full texts of the remaining articles were perused. Disagreements were resolved by consensus.

### Data Extraction and Quality Assessment

Two reviewers independently extracted the study characteristics using a predesigned standardized form. The primary outcome was the change from baseline in glycated hemoglobin (HbA_1c_) levels. Secondary efficacy endpoints of interest included the change from baseline in body weight, achievement of HbA_1c_ targets (<7.0% or ≤6.5%), and weight loss of at least 5% or 10%. We also examined the safety endpoints, including the number of hypoglycemic episodes, acute pancreatitis, diabetic retinopathy, malignant neoplasms, gastrointestinal adverse events, serious adverse events, cardiovascular events, cardiovascular death, and all-cause death. For efficacy outcomes, data for the approved doses of subcutaneous semaglutide (0.5 and 1 mg) or oral semaglutide (7 and 14 mg) administration were obtained. For safety outcomes, data for all doses of subcutaneous or oral semaglutide administration were obtained. The quality of the included RCTs was assessed using the Cochrane Collaboration’s Risk of Bias tool (Higgins et al., 2011). All disagreements were resolved by a third person.

### Data Analysis

Review Manager (RevMan 5.3, Nordic Cochrane Center, Copenhagen, Denmark) was used for all statistical analyses. Risk ratios (RRs) and weighted mean differences (WMDs) were separately calculated for dichotomous and continuous variables. All results were reported with their 95% confidence intervals (CIs). A *p-*value < 0.05 indicated a statistically significant difference. Pairwise meta-analyses were performed using random-effects models. Statistical heterogeneity across studies was estimated using the *I* ^*2*^ statistic, and the *I* ^*2*^ values > 75% indicated considerable heterogeneity (Higgins et al., 2003). For efficacy outcomes, data from different approved doses of subcutaneous semaglutide (0.5 and 1 mg) or oral semaglutide (7 and 14 mg) administration were analyzed among the subgroups. For safety outcomes, data for all doses of subcutaneous or oral semaglutide administration were combined. Given the heterogeneity of the comparator arm, we conducted separate meta-analyses for all outcomes in placebo- and active-controlled trials. Moreover, subgroup analyses for comparison of semaglutide with other GLP-1 RAs were also performed for efficacy outcomes.

## Results

### Search Results and Included Studies

The study selection process is illustrated in Supplementary Figure S1. Briefly, a total of 904 articles were retrieved in the initial electronic search. After meticulous screening for eligibility, 24 trials comprising 22,185 patients that met the inclusion criteria were included in the meta-analysis. The characteristics of the included studies are summarized in Supplementary Table S1. All trials were phase 3 RCTs that had reported their primary results between 2016 and 2021. These included the Semaglutide Unabated Sustainability in Treatment of Type 2 Diabetes (SUSTAIN) 1–10 trials, SUSTAIN China trial, Peptide Innovation for Early Diabetes Treatment (PIONEER) 1–10 trials, and three other studies.

Among the included studies on subcutaneous semaglutide administration, five trials investigated its efficacy and safety compared to placebo (Marso et al., 2016; Sorli et al., 2017; Rodbard et al., 2018; Zinman et al., 2019b; Davies et al., 2021); nine trials assessed its efficacy and safety versus other anti-diabetic drugs, including sitagliptin (Ahrén et al., 2017; Seino et al., 2018; Ji et al., 2020), canagliflozin (Lingvay et al., 2019), dulaglutide (Pratley et al., 2018), liraglutide (Capehorn et al., 2020), exenatide extended-release (Ahmann et al., 2018), insulin glargine (Aroda et al., 2017), and oral antihyperglycemic medications (Kaku et al., 2018). The sample size of these trials ranged from 302 in the SUSTAIN 9 study (Zinman et al., 2019b) to 3,297 in the SUSTAIN 6 study (Marso et al., 2016).

The studies on oral semaglutide administration included the following: six trials investigated its efficacy and safety compared to placebo (Aroda et al., 2019; Zinman et al., 2019a; Husain et al., 2019; Mosenzon et al., 2019; Pratley et al., 2019; Yamada et al., 2020); six trials compared its efficacy and safety with other anti-diabetic drugs, including sitagliptin (Pieber et al., 2019; Rosenstock et al., 2019), empagliflozin (Rodbard et al., 2019), dulaglutide (Yabe et al., 2020), and liraglutide (Pratley et al., 2019; Yamada et al., 2020). The sample size of these trials ranged from 243 in the PIONEER 9 study (Yamada et al., 2020) to 3,183 in the PIONEER 6 study (Husain et al., 2019).

Overall, most of the included studies had a low risk of bias evaluated according to the Cochrane Collaboration’s Risk of Bias tool, while some studies had a potential risk of bias because of their open-label design. The results of the risk of bias assessment are shown in Supplementary Figures S2, 3.

### Efficacy Assessment

#### Glycemic Control

**FIGURE 1:**
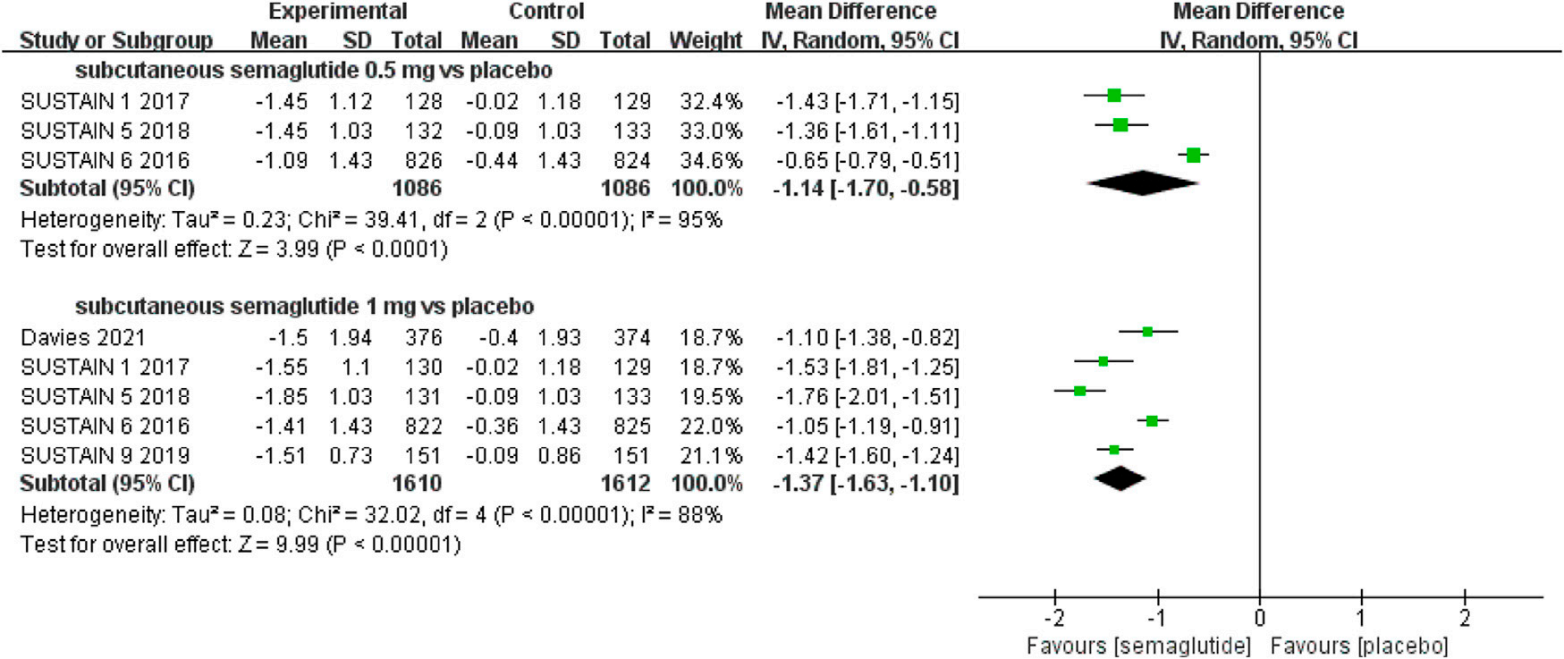
Weighted mean differences in change in HbA_1c_ (%) between the subcutaneous semaglutide and placebo-controlled arms.

**FIGURE 2:**
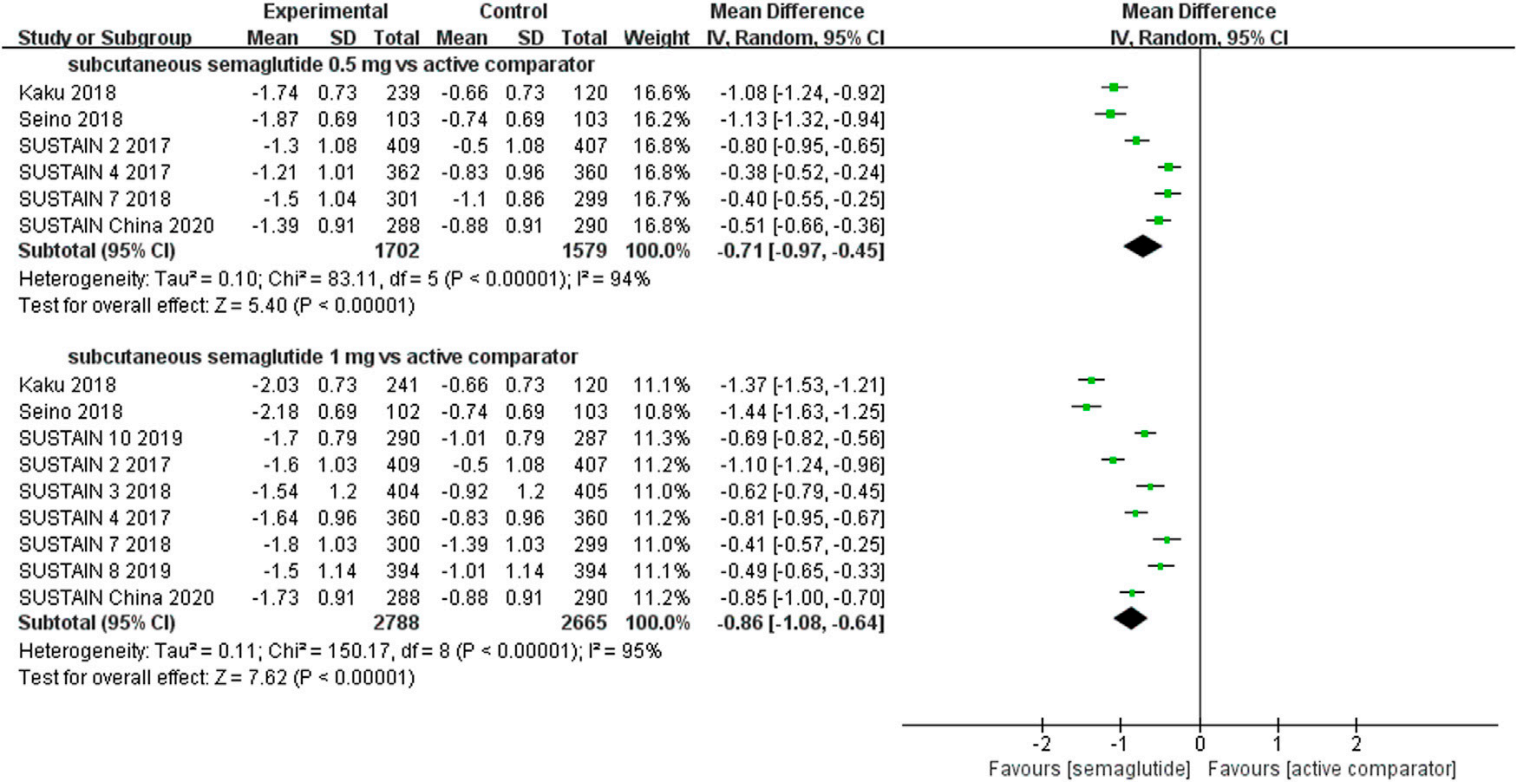
Weighted mean differences in change in HbA_1c_ (%) between the subcutaneous semaglutide and active-controlled arms.

**FIGURE 3:**
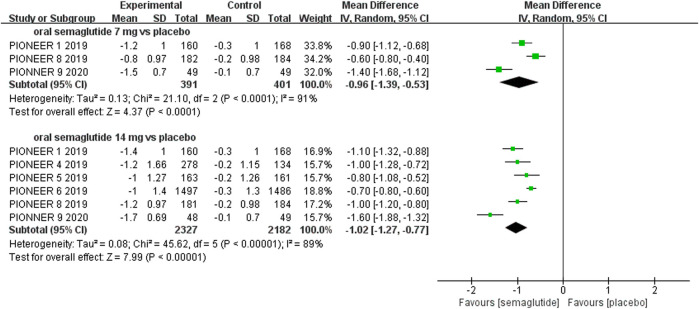
Weighted mean differences in change in HbA_1c_ (%) between the oral semaglutide and placebo-controlled arms.

**FIGURE 4:**
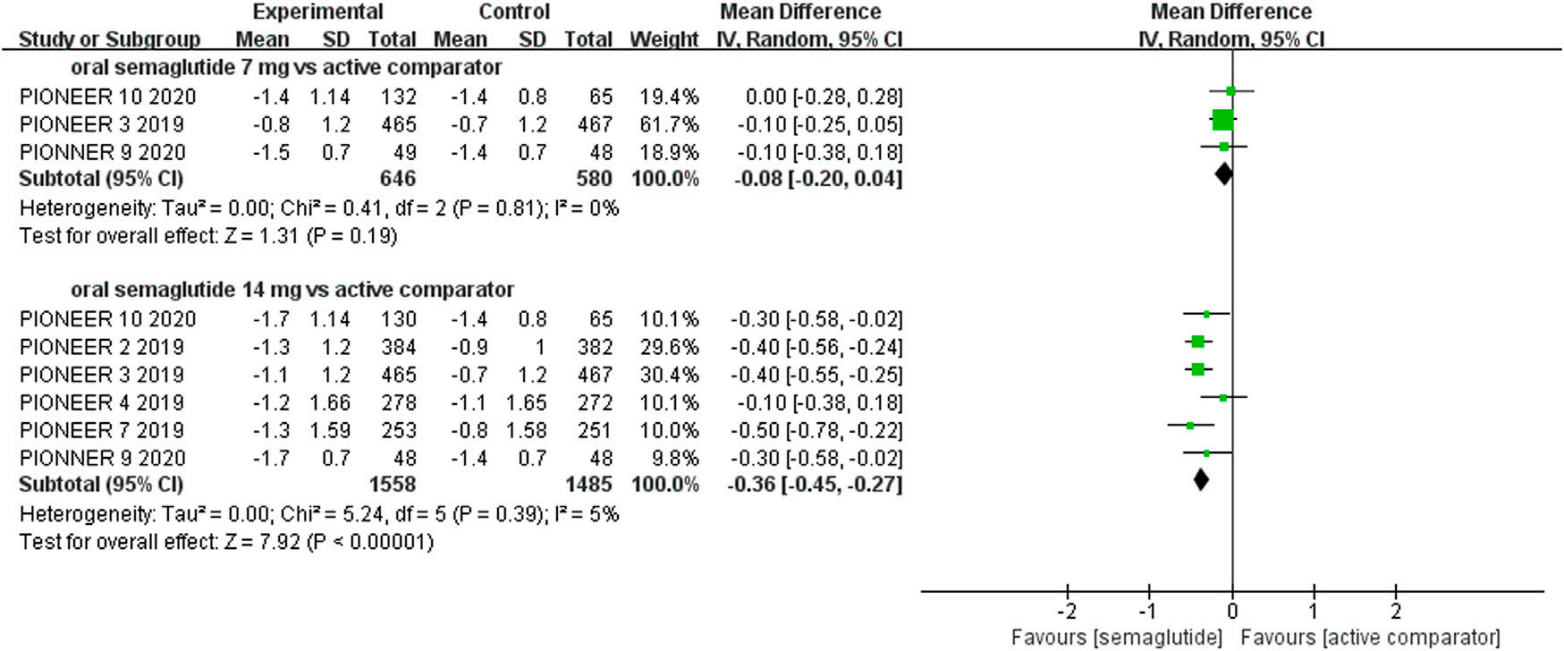
Weighted mean differences in change in HbA_1c_ (%) between the oral semaglutide and active-controlled arms.

The effects of subcutaneous and oral semaglutide administration on glycemic control are summarized in Figures 1–4, Supplementary Figures S4–11, and Supplementary Tables S2, 3. Taken together, the outcomes were in favor of subcutaneous and oral semaglutide administration over placebo and other anti-diabetic drugs. Subcutaneous semaglutide administration significantly reduced HbA_1c_ levels (WMD: −1.14%, 95% CI: −1.70 to −0.58; WMD: −1.37%, 95% CI: −1.63 to −1.10, for 0.5 and 1 mg, respectively) when compared with placebo, and its efficacy was also superior to active comparators (WMD: −0.71%, 95% CI: −0.97 to −0.45; WMD: −0.86%, 95% CI: −1.08 to −0.64, for 0.5 and 1 mg, respectively). Oral semaglutide administration significantly reduced HbA_1c_ levels (WMD: −0.96%, 95% CI: −1.39 to −0.53; WMD: −1.02%, 95% CI: −1.27 to −0.77, for 7 and 14 mg, respectively) when compared with placebo; oral administration of 14 mg of semaglutide also resulted in a greater reduction in HbA_1c_ levels when compared with active comparators (WMD: −0.36%, 95% CI: −0.45 to −0.27). Moreover, subcutaneous semaglutide (1 mg) and oral semaglutide (14 mg) administration were significantly more effective than other GLP-1 RAs in their extent of reduction in HbA_1c_ levels (WMD: −0.58%, 95% CI: −0.74 to –0.41; WMD: −0.23%, 95% CI: −0.39 to −0.07, respectively).

Furthermore, higher proportions of patients achieved the HbA_1c_ targets of <7.0% or ≤6.5% when semaglutide was administered subcutaneously (HbA_1c_ <7.0%: RR: 3.91, 95% CI: 2.16 to 7.11, and RR: 3.76, 95% CI: 2.60 to 5.42, for 0.5 and 1 mg, respectively; HbA_1c_ ≤6.5%: RR: 5.46, 95% CI: 3.25 to 9.16, and RR: 6.90, 95% CI: 4.07 to 11.69, for 0.5 and 1 mg, respectively) or orally (HbA_1c_ <7.0%: RR: 3.44, 95% CI: 1.86 to 6.35, and RR: 3.62, 95% CI: 2.50 to 5.25, for 7 and 14 mg, respectively; HbA_1c_ ≤6.5%: RR: 5.64, 95% CI: 1.80 to 17.66, and RR: 7.45, 95% CI: 3.75 to 14.81, for 7 and 14 mg, respectively), compared with placebo. Notably, subcutaneous semaglutide (HbA_1c_ <7.0%: RR: 1.75, 95% CI: 1.47 to 2.07, and RR: 1.81, 95% CI: 1.54 to 2.13, for 0.5 and 1 mg, respectively; HbA_1c_ ≤6.5%: RR: 2.52, 95% CI: 1.83 to 3.47, and RR: 2.80, 95% CI 2.15 to 3.65, for 0.5 and 1 mg, respectively) and oral semaglutide administration (HbA_1c_ <7.0%: RR: 1.27, 95% CI: 1.10 to 1.46, and RR: 1.53, 95% CI: 1.25 to 1.86, for 7 and 14 mg, respectively; HbA_1c_ ≤6.5%: RR: 1.44, 95% CI: 1.05 to 1.96, and RR: 1.90, 95% CI: 1.50 to 2.40, for 7 and 14 mg, respectively) also resulted in significantly higher numbers of patients achieving the targets of HbA_1c_ than other anti-diabetic drugs. Additionally, subcutaneous semaglutide (1 mg) and oral semaglutide (14 mg) administration were also superior to other GLP-1 RAs for the attainment of glycemic targets (HbA_1c_ <7.0%: RR: 1.51, 95% CI: 1.15 to 1.98, and RR: 1.31, 95% CI: 1.03 to 1.67, respectively; HbA_1c_ ≤6.5%: RR: 1.90, 95% CI: 1.35 to 2.66, and RR: 1.47, 95% CI: 1.17 to 1.84, respectively).

#### Weight Control

Patients who were given semaglutide either subcutaneously or orally lost more body weight than those in placebo- or active-controlled conditions. Compared with placebo, subcutaneous semaglutide administration of 0.5 and 1 mg reduced body weight by 2.73 kg (95% CI: 2.26–3.20) and 4.09 kg (95% CI: 3.55–4.63), respectively. Notably, the superiority of subcutaneously administered semaglutide over other anti-diabetic drugs in reducing body weight was also observed (WMD: −2.65 kg, 95% CI: −3.49 to −1.81, and WMD: −3.78 kg, 95% CI: −4.71 to −2.85, for 0.5 and 1 mg, respectively). Compared to placebo, the reduction in body weight was approximately 2.97 kg (95% CI: 2.19–3.74) with 14 mg oral semaglutide administration. Our results also showed the superiority of oral semaglutide administration over other anti-diabetic drugs in reducing body weight (WMD: −1.53 kg, 95% CI: −2.03 to −1.03, and WMD: −1.73 kg, 95% CI: −2.38 to −1.08, for 7 and 14 mg, respectively). Moreover, compared with other GLP-1 RAs, subcutaneous semaglutide (1 mg) and oral semaglutide (14 mg) administration yielded an improved reduction in body weight (WMD: −3.72 kg, 95% CI: −4.16 to −3.28, and WMD: −2.03 kg, 95% CI: −2.91 to −1.15, respectively).

In addition, both subcutaneous semaglutide (weight loss ≥5%: RR: 4.26, 95% CI: 2.83 to 6.42, and RR: 4.53, 95% CI: 2.08 to 9.89, for 0.5 and 1 mg, respectively; weight loss ≥10%: RR: 3.16, 95% CI: 1.38 to 7.28, and RR: 5.43, 95% CI: 2.98 to 9.89, for 0.5 and 1 mg, respectively) and oral semaglutide administration (weight loss ≥5%: RR: 3.20, 95% CI: 1.75 to 5.84, and RR: 4.01, 95% CI: 2.81 to 5.71, for 7 and 14 mg, respectively; weight loss ≥10%: RR: 9.42, 95% CI: 2.87 to 30.86, and RR: 8.99, 95% CI: 4.32 to 18.69, for 7 and 14 mg, respectively) were associated with higher proportions of patients achieving weight loss targets (≥5% or 10%) when compared with placebo. Similarly, subcutaneous semaglutide (weight loss ≥5%: RR: 3.93, 95% CI: 2.42 to 6.39, and RR: 3.67, 95% CI: 2.14 to 6.28, for 0.5 and 1 mg, respectively; weight loss ≥10%: RR: 4.56, 95% CI: 3.14 to 6.62, and RR: 5.65, 95% CI: 3.61 to 8.84, for 0.5 and 1 mg, respectively) and oral semaglutide administration (weight loss ≥5%: RR: 1.99, 95% CI 1.54 to 2.58, and RR: 2.10, 95% CI: 1.36 to 3.24, for 7 and 14 mg, respectively; weight loss ≥10%: RR: 2.59, 95% CI: 1.56 to 4.31, and RR: 2.27, 95% CI 1.75 to 2.95, for 7 and 14 mg, respectively) were also superior to active comparators.

**FIGURE 5:**
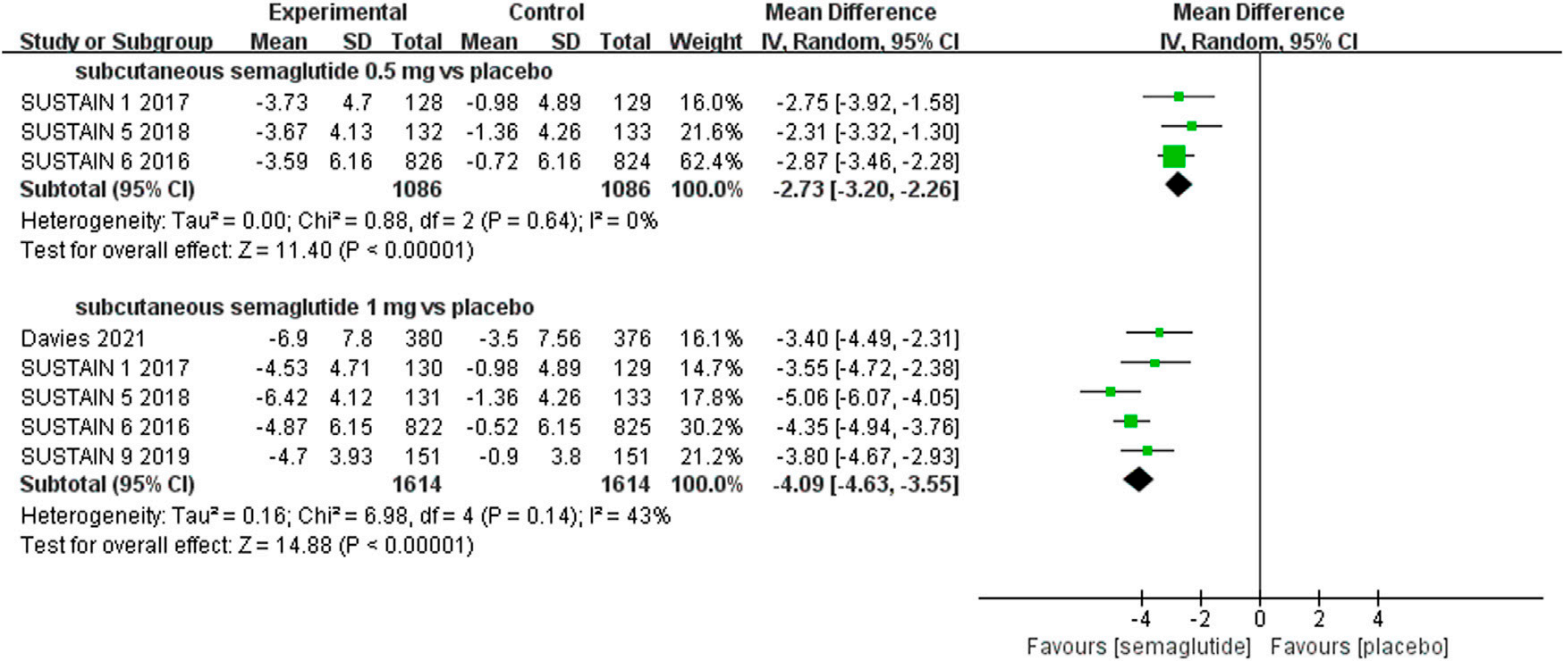
Weighted mean differences in change in body weight (kg) between the subcutaneous semaglutide and placebo-controlled arms.

**FIGURE 6:**
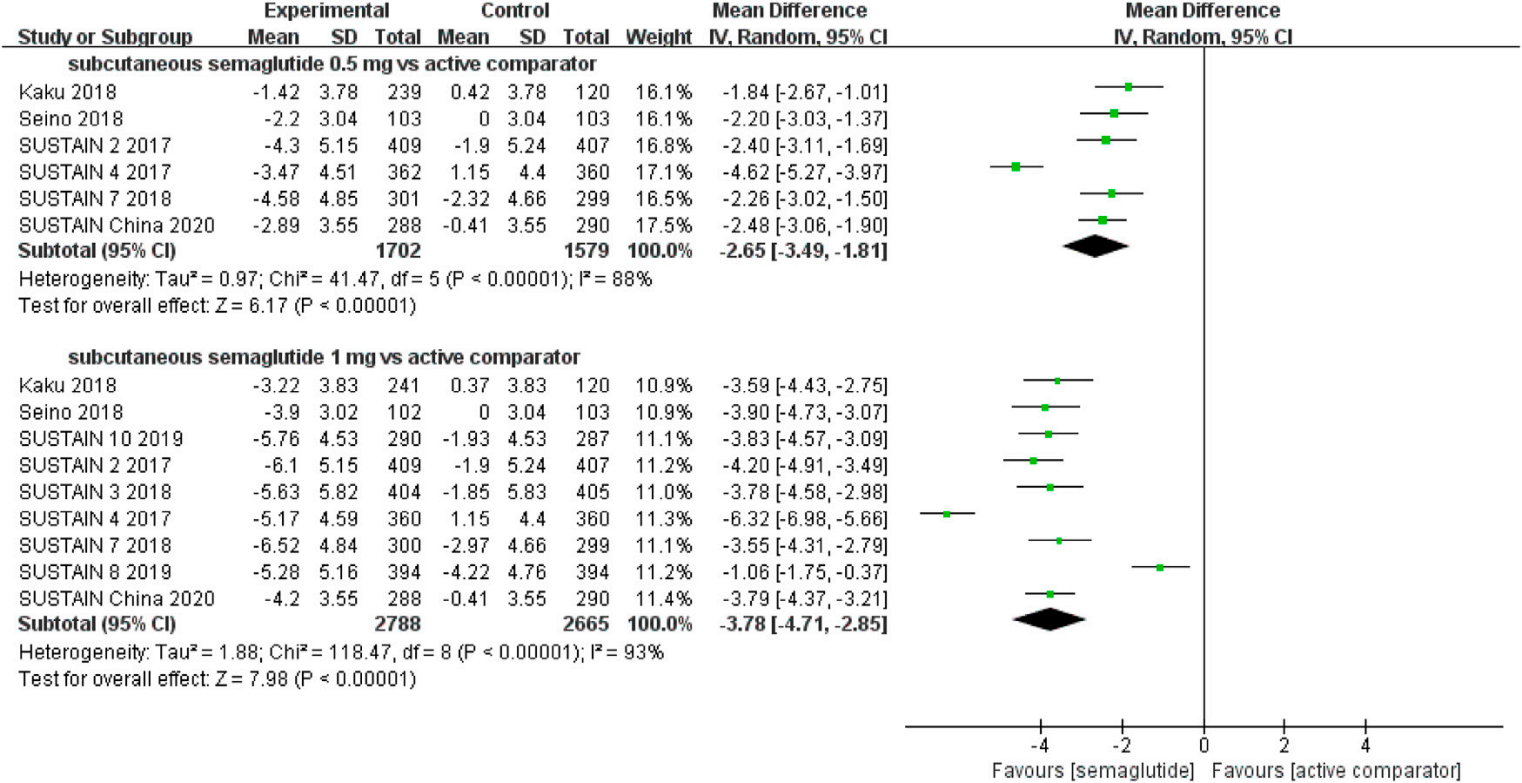
Weighted mean differences in change in body weight (kg) between the subcutaneous semaglutide and active-controlled arms.

**FIGURE 7:**
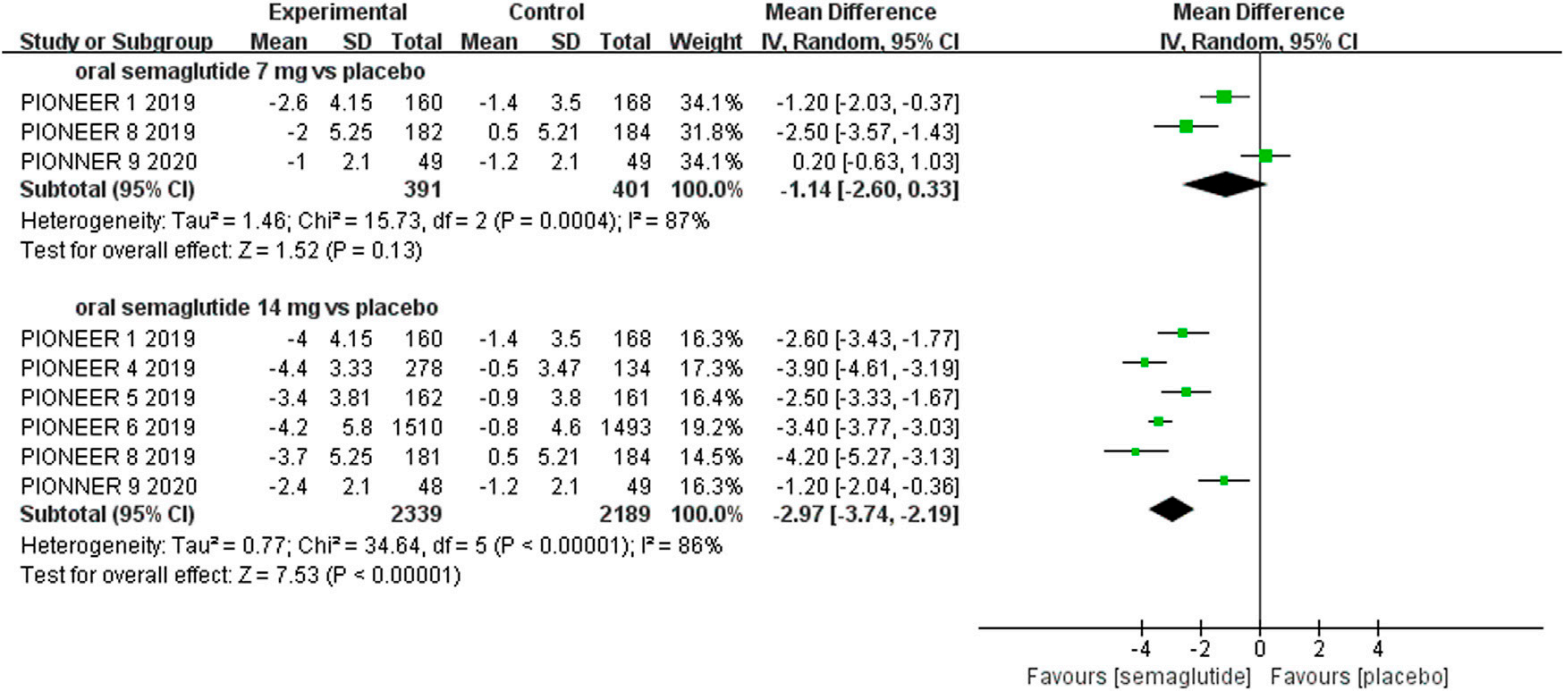
Weighted mean differences in change in body weight (kg) between the oral semaglutide and placebo-controlled arms.

**FIGURE 8:**
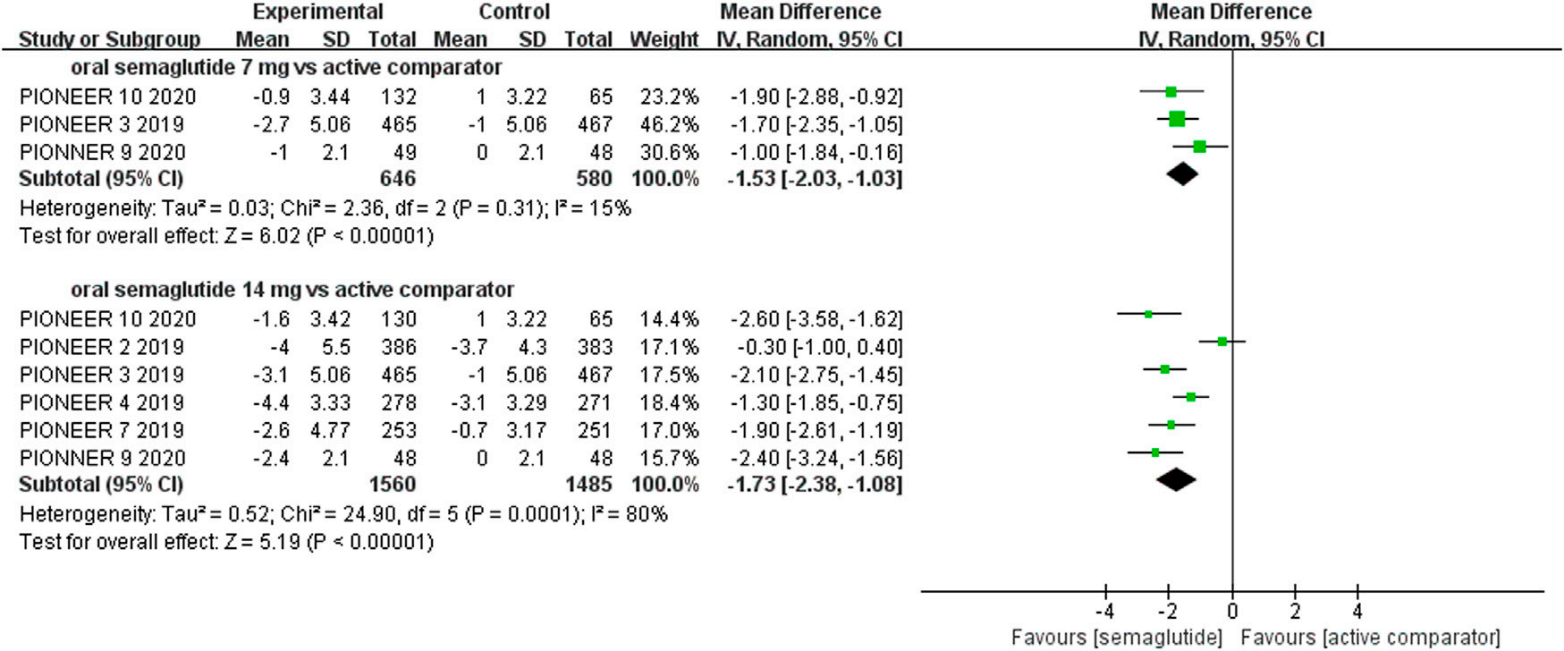
Weighted mean differences in change in body weight (kg) between the oral semaglutide and active-controlled arms.

Compared with other GLP-1 RAs, subcutaneous semaglutide (1 mg) and oral semaglutide (14 mg) administration also yielded an improved likelihood of attaining weight loss targets (weight loss ≥5%: RR: 1.92, 95% CI: 1.16 to 3.18, and RR: 3.09, 95% CI: 1.30 to 7.31, respectively; weight loss ≥10%: RR: 4.11, 95% CI: 3.09 to 5.48, and RR: 2.28, 95% CI: 1.40 to 3.73, respectively). These effects on weight control are presented in Figures 5–8, Supplementary Figures S12–19, and Supplementary Tables S2, 3.

### Safety Outcomes

Results for other safety endpoints are shown in Supplementary Tables S4, 5. Overall, the outcomes were either similar or better for subcutaneous and oral semaglutide administration than placebo and other anti-diabetic drugs. Compared with placebo and other anti-diabetic drugs, oral semaglutide administration did not increase the incidence of hypoglycemic events (RR: 1.29, 95% CI: 0.71 to 2.32, and RR: 0.74, 95% CI: 0.50 to 1.09, respectively). Compared with other anti-diabetic drugs, subcutaneous semaglutide administration was associated with a lower risk of hypoglycemia (RR: 0.73, 95% CI: 0.56–0.95), although no differences were observed when compared with placebo (RR: 1.36, 95% CI: 0.78–2.39).

Additionally, compared with placebo, subcutaneous semaglutide administration was associated with a lower risk of cardiovascular events (RR: 0.81, 95% CI: 0.66–0.98), while oral semaglutide administration reduced the risk of all-cause death (RR: 0.56, 95% CI: 0.35–0.90) and cardiovascular death (RR: 0.55, 95% CI: 0.31-0.98). However, no apparent differences were observed when they were compared with other antihyperglycemic medications. Moreover, pooled analyses of included trials also demonstrated that there were no significant differences in the risk of acute pancreatitis, diabetic retinopathy, and malignant neoplasms with subcutaneous and oral semaglutide administration compared to other anti-diabetic drugs and placebo. However, more patients who were given semaglutide either subcutaneously or orally experienced gastrointestinal adverse events (versus placebo: RR: 2.14, 95% CI: 1.61 to 2.85, and RR: 3.20, 95% CI: 2.58 to 3.97, respectively; versus other anti-diabetic drugs: RR: 2.28, 95% CI: 1.65 to 3.14, and RR: 1.98, 95% CI: 1.22 to 3.19, respectively), which were mainly of mild-to-moderate severity, as compared to those who were given placebo and other anti-diabetic drugs.

## Discussion

Semaglutide, a novel FDA-approved GLP-1 RA, has a positive effect in managing T2D, as demonstrated in the SUSTAIN and PIONEER trials. Here, we conducted a comprehensive meta-analysis with all the available data from phase 3 RCTs to investigate the efficacy and safety of subcutaneous and oral semaglutide administration, covering a broad scope of clinically relevant outcomes. In summary, our results suggested that, compared with placebo and other anti-diabetic drugs, subcutaneous and oral semaglutide administration resulted in significant reductions in HbA_1c_ levels, efficacious weight loss, no apparent increase in hypoglycemia, and no cardiovascular and other safety concerns except for non-serious gastrointestinal symptoms.

The results of a placebo-comparison meta-analysis of six GLP-1 RAs (Htike et al., 2017) show that this class of agents is associated with significant reductions in HbA_1c_ levels (ranging from −0.55 to −1.21%). In line with the study, our results suggested that subcutaneous and oral semaglutide administration could efficaciously reduce HbA_1c_ levels to a similar extent. The American Diabetes Association (ADA) recommends a reasonable target of a reduction of HbA_1c_ levels to <7.0% for most non-pregnant adults with T2D, which is also associated with a reduction in rates of microvascular complications and cardiovascular diseases (Riddle. et al., 2019b). Individuals at low risk for hypoglycemia and other adverse effects may reap more benefits of tight glycemic control, that is, an HbA_1c_ level of 6.5% or less (Riddle. et al., 2019b; Laiteerapong et al., 2019). Notably, the findings of our meta-analysis showed that subcutaneous and oral semaglutide outperformed the administration of placebo and other anti-diabetic drugs in achieving both the reasonable and stringent glycemic targets. In addition to providing effective glycemic control, the present study also demonstrated an overall superior effect on the absolute change in body weight and attainment of clinically significant weight loss by subcutaneous and oral semaglutide administration compared to placebo and other anti-diabetic drugs. Indeed, increasing evidence shows that weight loss of 3–5% results in substantial clinical health benefits for overweight and obese adults with multiple cardiovascular risk factors; more significant weight losses (e.g., 5–10%) produce greater benefits (Jensen et al., 2014). Accordingly, the ADA highly recommends a combination treatment of weight loss medication and lifestyle intervention to strengthen weight management for patients with T2D and body mass index >27 kg/m^2^ (Riddle. et al., 2019c).

Collectively, our study indicated that semaglutide had significantly greater benefits for glycemic control and weight management among T2D patients. Its potent therapeutic efficacy may be attributable to several underlying mechanisms of action. First, semaglutide is a long-acting GLP-1 RA. It can directly stimulate insulin secretion from pancreatic β-cells while suppressing glucagon release from pancreatic α-cells (Cornell, 2020). Specifically, the structure and pharmacokinetics of semaglutide are distinct from other GLP-1 RAs, as it shows 94% sequence homology with native GLP-1, sufficiently high GLP-1 receptor affinity, and exhibits an extended plasma half-life of approximately 1 week (Lau et al., 2015; Gallwitz and Giorgino, 2021). Second, semaglutide suppresses appetite and consequently leads to reduced energy intake through direct and secondary effects in the hypothalamus and area postrema of the brain, both of which are involved in appetite regulation and energy metabolism (Gabery et al., 2020). Third, from a pathophysiological standpoint, weight management and glycemic control seem to be mutually reinforcing. There is overwhelming evidence that obesity management helps mitigate fat-induced metabolic stress in the liver and pancreas and moderately improves insulin secretory capacity of pancreatic β-cells and insulin resistance (Steven et al., 2016; Taylor et al., 2019), thereby leading to a significant improvement in glycemic control and even remission of T2D (Schauer et al., 2016; Lean et al., 2018; Lean et al., 2019).

This systematic study substantially expanded on previous meta-analyses (Shi et al., 2018) and had strengths in several regards. First, we collated all data on subcutaneous and oral semaglutide administration from recent clinical studies to examine their efficacy and safety for the treatment of T2D across a wide range of glycemic control, weight management, and safety outcomes. The present meta-analysis included all the latest, well-designed, and multinational phase 3 RCTs, contributing to an up-to-date and robust evidence-based analysis. Moreover, for the individual approved doses of subcutaneous and oral semaglutide administration, we performed separate analyses to present the dose relatedness of efficacy. Subgroup analyses were also conducted based on different comparators (placebo, other widely used anti-diabetic drugs, and GLP-1 RAs). Furthermore, our study included clinically meaningful targets for glycemic control and weight loss as critical secondary outcomes. Finally, to confirm the cardiovascular safety of subcutaneous and oral semaglutide administration, we also evaluated their effects on cardiovascular events, cardiovascular death, and all-cause death. All these aspects further enhanced the clinical implications of this meta-analytic study, which could benefit physicians in making treatment-related decisions that suit the individual needs of the patients. Despite the aforementioned merits, there were some limitations of our study. First, aggregate trial-level data were utilized instead of patient-level data in this meta-analysis. Second, the number of included trials was limited (less than 10) for a single outcome; thus, we did not use funnel plots to assess the potential of publication bias. Third, because the pharmaceutical company supported all the included trials, sponsorship bias may exist in the current study.

In recent years, management measures for diabetes have extended beyond glycemic control alone. Owing to incremental therapeutic advances and the advent of new antihyperglycemic medications, obesity treatment and cardiovascular risk management have become essential in establishing the standards of medical care in diabetes (Riddle. et al., 2019e). Our study suggested that subcutaneous and oral semaglutide administration outperformed other widely used anti-diabetic drugs for glycemic control and weight loss. Currently, semaglutide is the only approved GLP-1 RA that can be administered by both oral and subcutaneous routes. Due to the relatively low oral bioavailability of peptide medications, larger doses of orally administered semaglutide are needed to attain plasma concentrations comparable with those attained via subcutaneous injection (Rosenstock et al., 2019). Although the two formulations of semaglutide differ in their absorption profiles, once absorbed, no apparent differences are observed in the pharmacokinetic properties and exposure-response relationships in their efficacy and safety (Davies et al., 2017; Granhall et al., 2019; Overgaard et al., 2020; Meier, 2021).

Current international guidelines recommend GLP-1 RAs with proven cardiovascular benefits for patients with T2D and established cardiovascular disease or risk factors to reduce cardiovascular events (Riddle. et al., 2019d; Cosentino et al., 2020). In this study, when compared with placebo, subcutaneous semaglutide administration appeared to moderately reduce cardiovascular events, while oral semaglutide administration was associated with a lower risk of cardiovascular and all-cause mortality. Notably, the two cardiovascular outcomes noninferiority trials enrolling patients with T2D at high cardiovascular risk, the SUSTAIN 6 trial (Marso et al., 2016) and the PIONEER 6 trial (Husain et al., 2019), contributed to the majority of the data used in our analyses. Subsequent large-scale studies with longer durations are needed to validate the promising superiority and independent cardiovascular benefits of semaglutide for T2D patients and other populations. Furthermore, although, in this study, phase 3 clinical trials with relatively short follow-up periods demonstrated a neutral effect of subcutaneous and oral semaglutide administration on diabetic retinopathy, the long-term effect of semaglutide on diabetic eye disease still requires further investigation in the post-marketing surveillance phase. In addition, similar to other GLP-1 RAs, subcutaneous and oral semaglutide administration may also induce mild-to-moderate gastrointestinal system disorders, which remains a safety concern and serves as the primary reason for discontinuation of treatment, albeit with a standard dose-escalation procedure (Smits and Van Raalte, 2021). Consequently, the strategies to remit the negative influence of gastrointestinal events associated with semaglutide and other GLP-1 RAs on patient adherence are necessary and need to be addressed in future research.

## Conclusion

The prominent capacity of subcutaneous and oral semaglutide administration to improve glycemic control and weight management with no key safety concerns represents a major therapeutic advance. In conclusion, our findings provided robust evidence to support subcutaneous and oral semaglutide administration for the clinical treatment of patients with T2D, especially among those with concomitant obesity.

## Data Availability

The original contributions presented in the study are included in the article/Supplementary Material; further inquiries can be directed to the corresponding author.
