# Supplementary figures and images for: Efficacy and Safety of Subcutaneous and Oral Semaglutide Administration in Patients With Type 2 Diabetes: A Meta-Analysis
Source: Front Pharmacol. 2021 Oct 6;12:695182. doi: 10.3389/fphar.2021.695182 (PMC8526557; doi:10.3389/fphar.2021.695182)

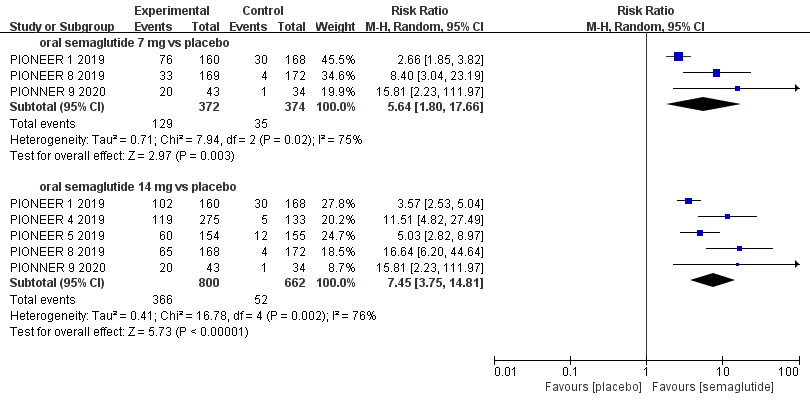

Supplement: Supplementary file 1 [file DataSheet1.zip › supplementary material/Figure S10. RRs for achieving HbA1c í▄ 6.5% oral semaglutide vs placebo.png]

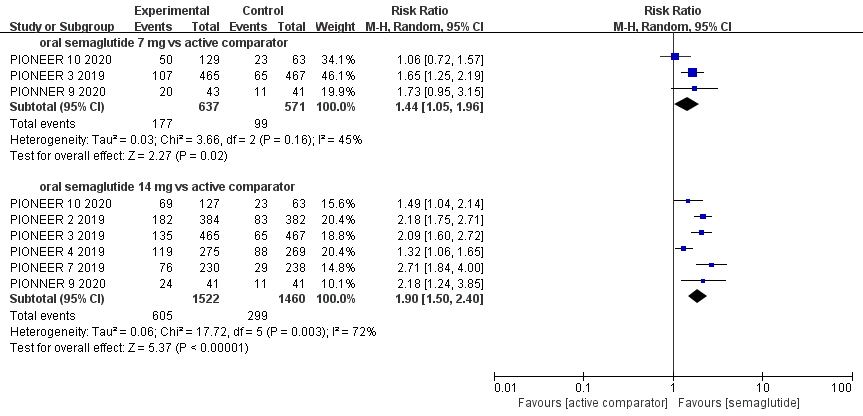

Supplement: Supplementary file 1 [file DataSheet1.zip › supplementary material/Figure S11. RRs for achieving HbA1c í▄ 6.5% oral semaglutide vs active comparator.png]

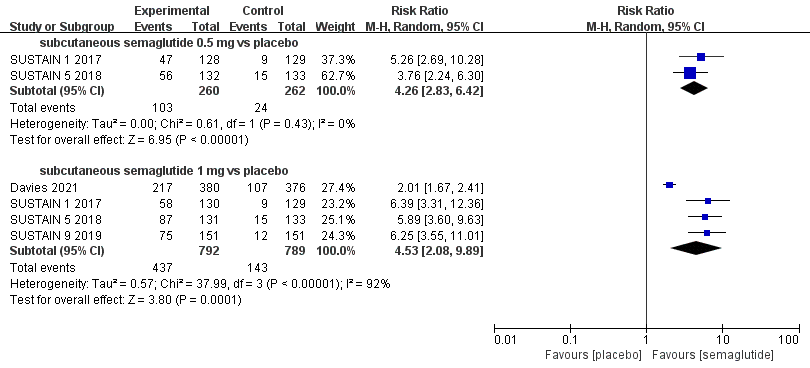

Supplement: Supplementary file 1 [file DataSheet1.zip › supplementary material/Figure S12. RRs for achieving weight loss í▌ 5% subcutaneous semaglutide vs placebo.png]

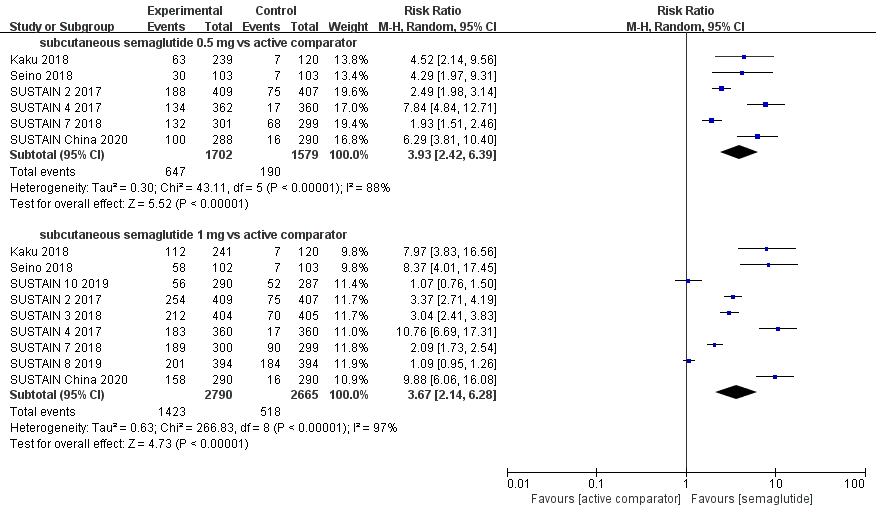

Supplement: Supplementary file 1 [file DataSheet1.zip › supplementary material/Figure S13. RRs for achieving weight loss í▌ 5% subcutaneous semaglutide vs active comparator.png]

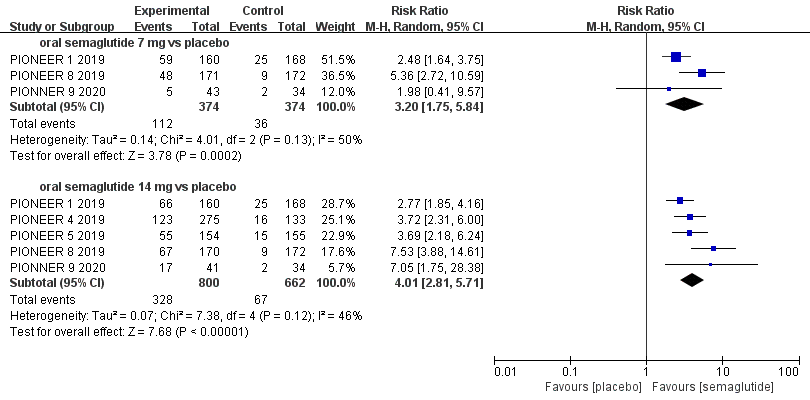

Supplement: Supplementary file 1 [file DataSheet1.zip › supplementary material/Figure S14. RRs for achieving weight loss í▌ 5% oral semaglutide vs placebo.png]

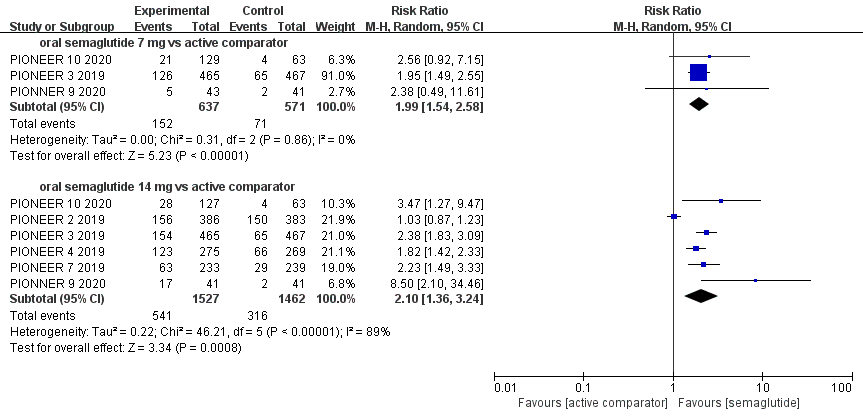

Supplement: Supplementary file 1 [file DataSheet1.zip › supplementary material/Figure S15. RRs for achieving weight loss í▌ 5% oral semaglutide vs active comparator.png]

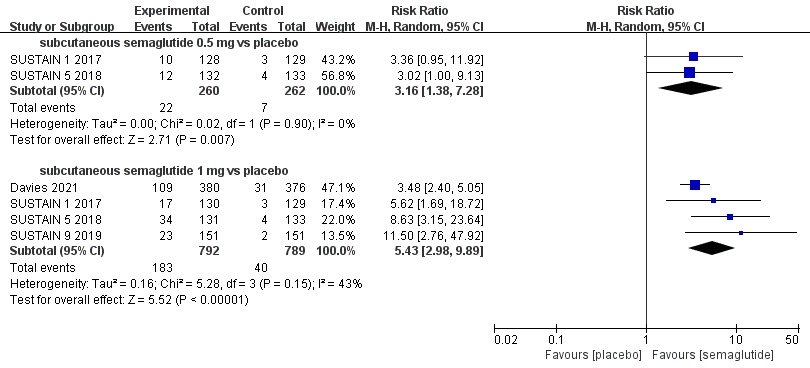

Supplement: Supplementary file 1 [file DataSheet1.zip › supplementary material/Figure S16. RRs for achieving weight loss í▌ 10% subcutaneous semaglutide vs placebo.png]

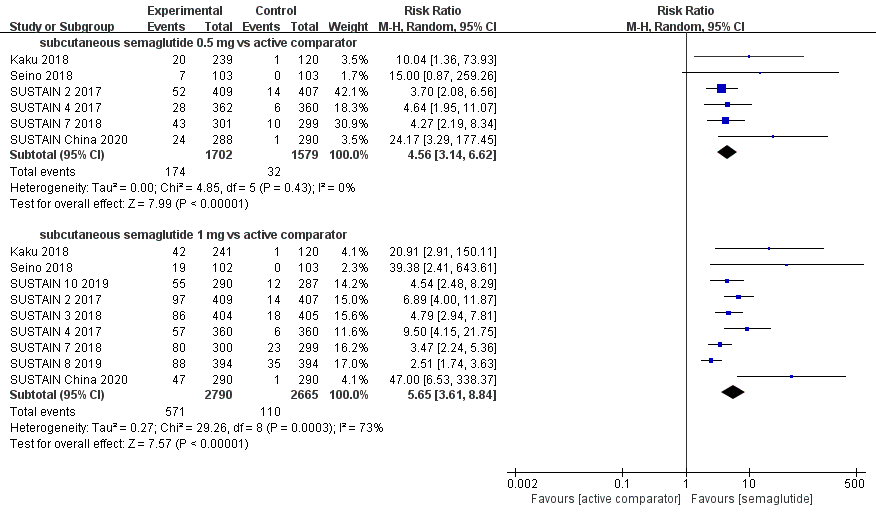

Supplement: Supplementary file 1 [file DataSheet1.zip › supplementary material/Figure S17. RRs for achieving weight loss í▌ 10% subcutaneous semaglutide vs active comparator.png]

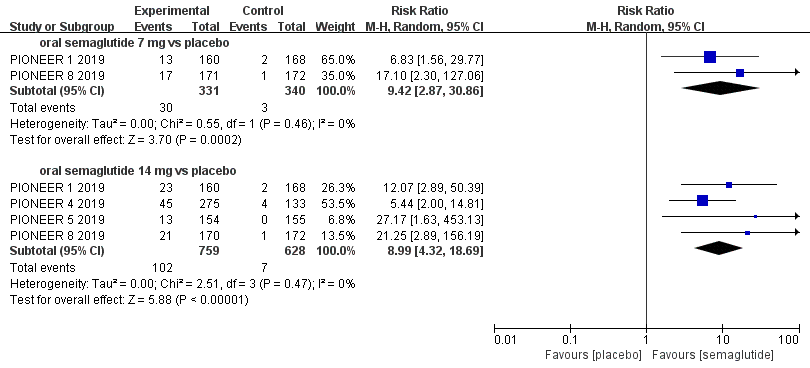

Supplement: Supplementary file 1 [file DataSheet1.zip › supplementary material/Figure S18. RRs for achieving weight loss í▌ 10% oral semaglutide vs placebo.png]

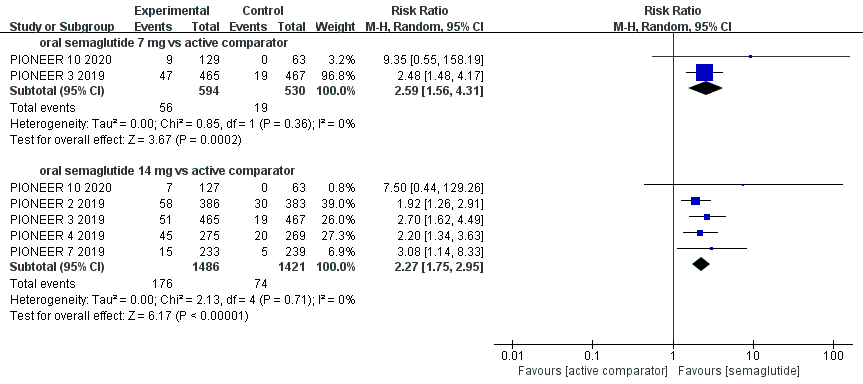

Supplement: Supplementary file 1 [file DataSheet1.zip › supplementary material/Figure S19. RRs for achieving weight loss í▌ 10% oral semaglutide vs active comparator.png]

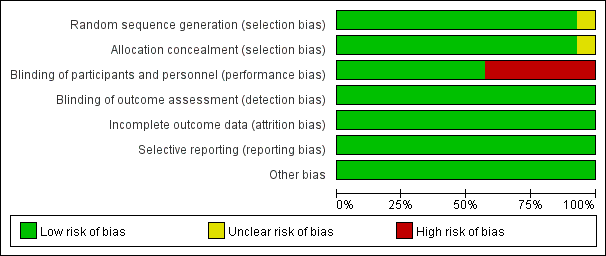

Supplement: Supplementary file 1 [file DataSheet1.zip › supplementary material/Figure S2. Risk of bias graph for RCTs of subcutaneous semaglutide administration.png]

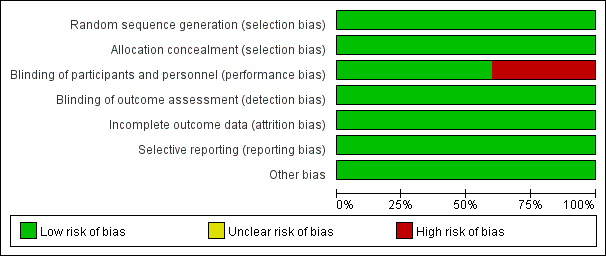

Supplement: Supplementary file 1 [file DataSheet1.zip › supplementary material/Figure S3. Risk of bias graph for RCTs of oral semaglutide administration.png]

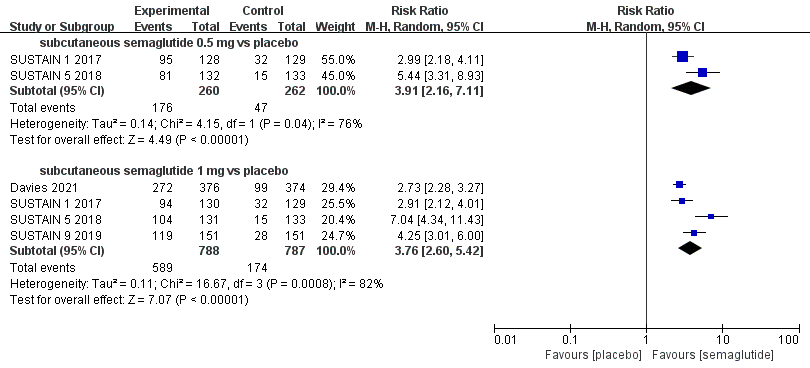

Supplement: Supplementary file 1 [file DataSheet1.zip › supplementary material/Figure S4. RRs for achieving HbA1c ú╝7.0% subcutaneous semaglutide vs placebo.png]

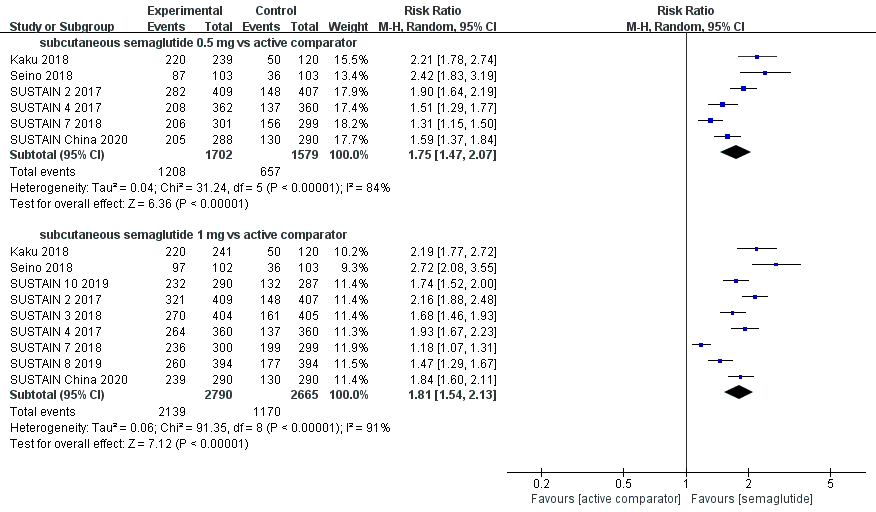

Supplement: Supplementary file 1 [file DataSheet1.zip › supplementary material/Figure S5. RRs for achieving HbA1c ú╝7.0% subcutaneous semaglutide vs active comparator.png]

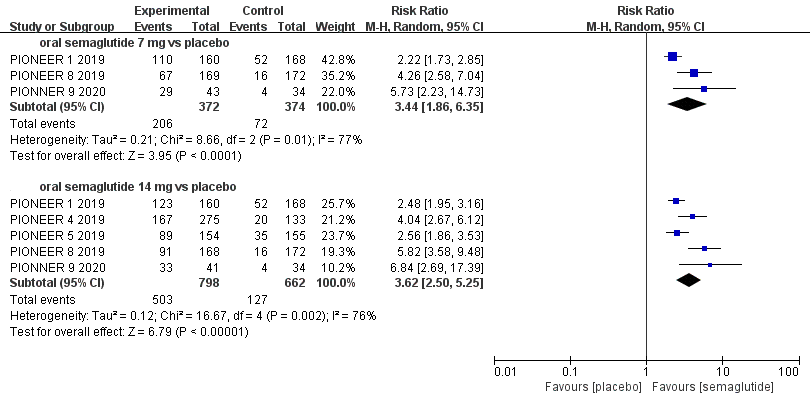

Supplement: Supplementary file 1 [file DataSheet1.zip › supplementary material/Figure S6. RRs for achieving HbA1c ú╝7.0% oral semaglutide vs placebo.png]

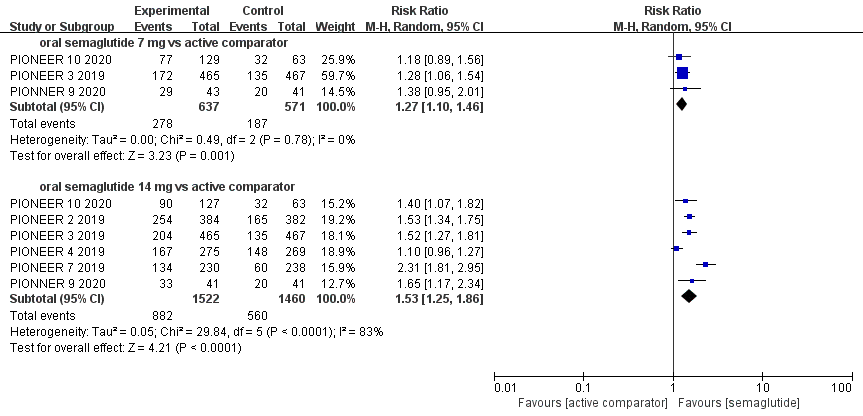

Supplement: Supplementary file 1 [file DataSheet1.zip › supplementary material/Figure S7. RRs for achieving HbA1c ú╝7.0% oral semaglutide vs active comparator.png]

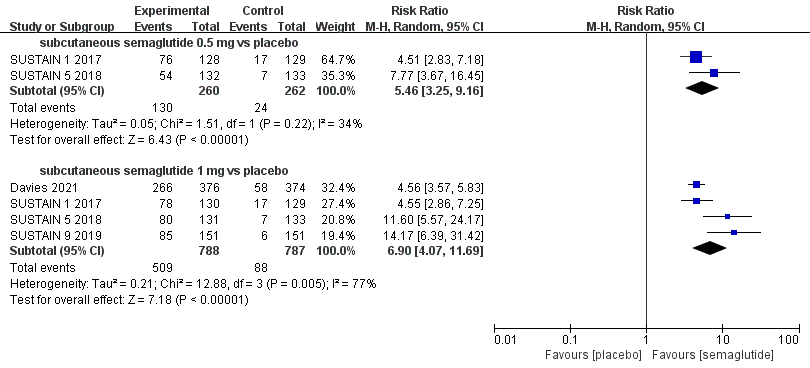

Supplement: Supplementary file 1 [file DataSheet1.zip › supplementary material/Figure S8. RRs for achieving HbA1c í▄ 6.5% subcutaneous semaglutide vs placebo.png]

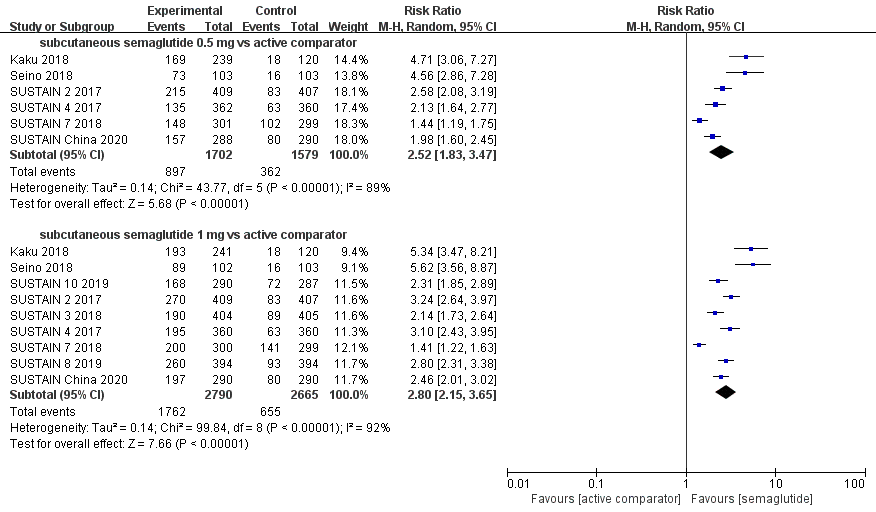

Supplement: Supplementary file 1 [file DataSheet1.zip › supplementary material/Figure S9. RRs for achieving HbA1c í▄ 6.5% subcutaneous semaglutide vs active comparator.png]
