# Supplementary material for: Efficacy and Safety of Subcutaneous and Oral Semaglutide Administration in Patients With Type 2 Diabetes: A Meta-Analysis
Source: Front Pharmacol. 2021 Oct 6;12:695182. doi: 10.3389/fphar.2021.695182 (PMC8526557; doi:10.3389/fphar.2021.695182)
Supplement: Supplementary file 1 [file DataSheet1.zip › supplementary material/Figure S1. PRISMA 2009 flow diagram.doc]

**FIGURE S1 PRISMA 2009 Flow Diagram**

**Screening**

**Included**

**Eligibility**

**Identification**

Records identified through

database searching (n = 904):

the Cochrane Library (n = 318)	Embase (n = 387) PubMed (n = 199)

Additional records identified through other sources
(n = 0 )

Records after duplicates removed
(n = 637 )

Records screened
(n = 637)

Records excluded
(n = 607):

Reviews and meta-analyses (n = 45)

Irrelevant studies

(n = 492)

Trial registering records (n = 53)

Conference abstract (n = 17)

Full-text articles assessed for eligibility
(n = 30)

Full-text articles excluded, with reasons
(n = 6 ):

Study design papers (n = 2)

Phase 1 or 2 study

(n = 4)

Studies included in qualitative synthesis
(n = 24)

Studies included in quantitative synthesis (meta-analysis)
(n = 24)
