# Supplementary material for: Efficacy and Safety of Subcutaneous and Oral Semaglutide Administration in Patients With Type 2 Diabetes: A Meta-Analysis
Source: Front Pharmacol. 2021 Oct 6;12:695182. doi: 10.3389/fphar.2021.695182 (PMC8526557; doi:10.3389/fphar.2021.695182)
Supplement: Supplementary file 1 [file DataSheet1.zip › supplementary material/Search strategies in three databases.doc]

Search strategy in PubMed.

Block 1: type 2 diabetes mellitus

#1 “Diabetes Mellitus, Type 2”[Mesh] OR “diabetes mellitus type 2”[tiab] OR “type 2 diabetes mellitus”[tiab] OR “type 2 diabetes”[tiab] OR “T2D”[tiab] OR “T2DM”[tiab] OR “non-insulin-dependent diabetes mellitus” [tiab] OR NIDDM[tiab]

Block 2: semaglutide

#2 (semaglutide[tiab] OR Ozempic[tiab] OR Rybelsus[tiab]) AND (oral[tiab] OR orally[tiab] OR subcutaneously[tiab] OR subcutaneous[tiab] OR once-weekly[tiab] OR once-daily[tiab])

Block 3: RCT-filter

#3 “randomized controlled trial”[pt] OR “controlled clinical trial”[pt] OR randomized[tiab] OR placebo[tiab] OR “drug therapy”[sh] OR randomly[tiab] OR trial[tiab] OR groups[tiab]

Block 4:

#4	#1 AND #2 AND #3

Search strategy in Embase.

Block 1: type 2 diabetes mellitus

#1 ('non insulin dependent diabetes mellitus'/exp OR 'type 2 diabetes mellitus':ti,ab OR 'diabetes mellitus type 2':ti,ab OR 'type 2 diabetes':ti,ab OR T2D:ti,ab OR T2DM:ti,ab OR NIDDM:ti,ab)

Block 2: semaglutide

#2 (semaglutide:ti,ab OR Ozempic:ti,ab OR Rybelsus:ti,ab) AND (oral:ti,ab OR orally:ti,ab OR subcutaneously:ti,ab OR subcutaneous:ti,ab OR once-weekly:ti,ab OR once-daily:ti,ab)

Block 3: RCT-filter

#3 ('clinical':ti,ab AND 'trial':ti,ab) OR 'clinical trial'/exp OR random* OR 'drug therapy':lnk

Block 4:

#4	#1 AND #2 AND #3

Search strategy in the Cochrane Library.

Block 1: type 2 diabetes mellitus

#1 ((Diabetes Mellitus, Type 2) OR (diabetes mellitus type 2) OR (type 2 diabetes mellitus) OR (type 2 diabetes) OR T2D OR T2DM OR (non-insulin-dependent diabetes mellitus) OR NIDDM) in Title Abstract Keyword

Block 2: semaglutide

#2 ((semaglutide OR Ozempic OR Rybelsus) AND (oral OR orally OR subcutaneously OR subcutaneous OR once-weekly OR once-daily)) in Title Abstract Keyword

Block 3:

#3 #2 AND #3

Abbreviations: NIDDM, non-insulin-dependent diabetes mellitus; T2D, type 2 diabetes; T2DM, type 2 diabetes mellitus.
