# Supplementary material for: Efficacy and Safety of Subcutaneous and Oral Semaglutide Administration in Patients With Type 2 Diabetes: A Meta-Analysis
Source: Front Pharmacol. 2021 Oct 6;12:695182. doi: 10.3389/fphar.2021.695182 (PMC8526557; doi:10.3389/fphar.2021.695182)
Supplement: Supplementary file 1 [file DataSheet1.zip › supplementary material/Table S1. Characteristics of included studies.doc]

**Table S1. Characteristics of included studies**

| **Study ID** | **Background treatment** | **Intervention and comparison** | **Patients**  **(N)** | **Percentage**  **of patients completing treatment (%)** | **Percentage**  **of men (%)** | **Mean age (years)** | **Mean baseline HbA_1c_ (%)** | **Mean baseline weight (kg)** | **Mean duration of diabetes (years)** | **Treatment duration (weeks)** |
| --- | --- | --- | --- | --- | --- | --- | --- | --- | --- | --- |
| Davies  2021 | Lifestyle intervention | Subcutaneous semaglutide 1 mg | 403 | 87.8 | 49.6 | 56 | 8.1 | 99 | 7.7 | 68 |
|  |  | Placebo | 403 | 86.1 | 52.9 | 55 | 8.1 | 100.5 | 8.2 |  |
| Kaku 2018 | Diet and exercise + OAD | Subcutaneous semaglutide 0.5 mg | 239 | 93.7 | 69.5 | 58.0 | 8.0 | 71.0 | 8.1 | 56 |
|  |  | Subcutaneous semaglutide 1 mg | 241 | 85.9 | 72.2 | 58.7 | 8.1 | 71.7 | 9.4 |  |
|  |  | One additional OAD | 120 | 94.2 | 74.2 | 59.2 | 8.1 | 72.2 | 9.3 |  |
| Seino 2018 | Diet and exercise | Subcutaneous semaglutide 0.5 mg | 103 | 97.1 | 76.6 | 58.8 | 8.2 | 67.8 | 8.0 | 30 |
|  |  | Subcutaneous semaglutide 1 mg | 102 | 85.3 | 73.5 | 58.1 | 8.0 | 70.8 | 7.8 |  |
|  |  | Sitagliptin 100 mg | 103 | 97.1 | 78.6 | 57.9 | 8.2 | 69.4 | 8.1 |  |
| SUSTAIN 1 2017 | Diet and exercise | Subcutaneous semaglutide 0.5 mg | 128 | 86.7 | 47 | 54.6 | 8.1 | 89.8 | 4.8 | 30 |
|  |  | Subcutaneous semaglutide 1 mg | 130 | 87.7 | 62 | 52.7 | 8.1 | 96.9 | 3.6 |  |
|  |  | Placebo | 129 | 89.1 | 54 | 53.9 | 8.0 | 89.1 | 4.1 |  |
| SUSTAIN 2 2017 | Metformin ± pioglitazone/rosiglitazone | Subcutaneous semaglutide 0.5 mg | 409 | 87.0 | 51 | 54.8 | 8.0 | 89.9 | 6.4 | 56 |
|  |  | Subcutaneous semaglutide 1 mg | 409 | 85.1 | 50 | 56.0 | 8.0 | 89.2 | 6.7 |  |
|  |  | Sitagliptin 100 mg | 407 | 92.1 | 51 | 54.6 | 8.2 | 89.3 | 6.6 |  |
| SUSTAIN 3 2018 | Metformin ± thiazolidinediones/sulfonylureas | Subcutaneous semaglutide 1 mg | 404 | 79.7 | 54.2 | 56.4 | 8.4 | 96.2 | 9.0 | 56 |
|  |  | Exenatide ER 2 mg | 405 | 79.0 | 56.3 | 56.7 | 8.3 | 95.4 | 9.4 |  |
| SUSTAIN 4 2017 | Metformin ± sulfonylureas | Subcutaneous semaglutide 0.5 mg | 362 | 86.5 | 54 | 56.5 | 8.1 | 93.7 | 7.8 | 30 |
|  |  | Subcutaneous semaglutide 1 mg | 360 | 84.7 | 51 | 56.7 | 8.3 | 94.0 | 9.3 |  |
|  |  | Insulin glargine | 360 | 92.8 | 54 | 56.2 | 8.1 | 92.6 | 8.6 |  |
| SUSTAIN 5 2018 | Basal insulin ± metformin | Subcutaneous semaglutide 0.5 mg | 132 | 89.4 | 56.1 | 59.1 | 8.4 | 92.7 | 12.9 | 30 |
|  |  | Subcutaneous semaglutide 1 mg | 131 | 87.8 | 58.8 | 58.5 | 8.3 | 92.5 | 13.7 |  |
|  |  | Placebo | 133 | 90.2 | 53.4 | 58.8 | 8.4 | 89.9 | 13.3 |  |
| SUSTAIN 6 2016 | < 2 OADs ± basal/premixed insulin | Subcutaneous semaglutide 0.5 mg | 826 | 80.1 | 59.9 | 64.6 | 8.7 | 91.8 | 14.3 | 104 |
|  |  | Subcutaneous semaglutide 1 mg | 822 | 77.4 | 63.0 | 64.7 | 8.7 | 92.9 | 14.1 |  |
|  |  | Placebo 0.5 mg | 824 | 81.7 | 58.5 | 64.8 | 8.7 | 91.8 | 14.0 |  |
|  |  | Placebo 1 mg | 825 | 80.7 | 61.5 | 64.4 | 8.7 | 91.9 | 13.2 |  |
| SUSTAIN 7 2018 | Metformin | Subcutaneous semaglutide 0.5 mg | 301 | 84.4 | 56 | 56 | 8.3 | 96.4 | 7.7 | 40 |
|  |  | Subcutaneous semaglutide 1 mg | 300 | 83.0 | 54 | 55 | 8.2 | 95.5 | 7.3 |  |
|  |  | Dulaglutide 0.75 mg | 299 | 91.0 | 54 | 55 | 8.2 | 95.6 | 7.0 |  |
|  |  | Dulaglutide 1.5 mg | 299 | 88.0 | 57 | 56 | 8.2 | 93.4 | 7.6 |  |
| SUSTAIN 8 2019 | Metformin | Subcutaneous semaglutide 1 mg | 394 | 83.8 | 57 | 55.7 | 8.3 | 90.6 | 7.5 | 52 |
|  |  | Canagliflozin 300 mg | 394 | 87.1 | 51 | 57.5 | 8.2 | 89.8 | 7.2 |  |
| SUSTAIN 9 2019 | An SGLT-2 inhibitor ±  sulphonylurea/metformin | Subcutaneous semaglutide 1 mg | 151 | 84.8 | 58.9 | 57.5 | 8.0 | 89.6 | 9.8 | 30 |
|  |  | Placebo | 151 | 92.1 | 57.6 | 56.6 | 8.1 | 93.8 | 9.6 |  |
| SUSTAIN 10 2019 | OADs | Subcutaneous semaglutide 1 mg | 290 | 85.9 | 55.2 | 60.1 | 8.2 | 96.6 | 9.6 | 30 |
|  |  | Liraglutide 1.2 mg | 287 | 90.9 | 58.2 | 58.9 | 8.3 | 97.2 | 8.9 |  |
| SUSTAIN China 2020 | Metformin | Subcutaneous semaglutide 0.5 mg | 288 | 87.2 | 55.6 | 53.0 | 8.1 | 77.6 | 6.3 | 30 |
|  |  | Subcutaneous semaglutide 1 mg | 290 | 84.1 | 53.1 | 53.0 | 8.1 | 76.1 | 6.7 |  |
|  |  | Sitagliptin 100 mg | 290 | 94.1 | 63.8 | 53.1 | 8.1 | 75.5 | 6.1 |  |
| PIONEER 1 2019 | Diet and exercise | Oral semaglutide 3 mg | 175 | 93.1 | 50.9 | 55 | 7.9 | 86.9 | 3.8 | 26 |
|  |  | Oral semaglutide 7 mg | 175 | 89.7 | 53.1 | 56 | 8.0 | 89.0 | 3.6 |  |
|  |  | Oral semaglutide 14 mg | 175 | 86.3 | 49.1 | 54 | 8.0 | 88.1 | 3.4 |  |
|  |  | Placebo | 178 | 89.3 | 50.0 | 54 | 7.9 | 88.6 | 3.4 |  |
| PIONEER 2 2019 | Metformin | Oral Semaglutide 14 mg | 411 | 82.5 | 50.1 | 57 | 8.1 | 91.9 | 7.2 | 52 |
|  |  | Empagliflozin 25 mg | 410 | 89.0 | 51.0 | 58 | 8.1 | 91.3 | 7.7 |  |
| PIONEER 3 2019 | Metformin ± sulfonylurea | Oral semaglutide 3 mg | 466 | 83.3 | 54.5 | 58 | 8.3 | 91.6 | 8.4 | 78 |
|  |  | Oral semaglutide 7 mg | 465 | 85.2 | 52.7 | 58 | 8.4 | 91.3 | 8.3 |  |
|  |  | Oral semaglutide 14 mg | 465 | 80.9 | 53.1 | 57 | 8.3 | 91.2 | 8.7 |  |
|  |  | Sitagliptin 100 mg | 467 | 86.9 | 51.0 | 58 | 8.3 | 90.9 | 8.8 |  |
| PIONEER 4 2019 | Metformin ± an SGLT-2 inhibitor | Oral semaglutide 14 mg | 285 | 84.6 | 52 | 56 | 8.0 | 92.9 | 7.8 | 52 |
|  |  | Liraglutide 1.8 mg | 284 | 87.3 | 52 | 56 | 8.0 | 95.5 | 7.3 |  |
|  |  | Placebo | 142 | 88.0 | 52 | 57 | 7.9 | 93.2 | 7.8 |  |
| PIONEER 5 2019 | Metformin or sulfonylurea, or both, or basal insulin ± metformin | Oral semaglutide 14 mg | 163 | 81.6 | 51 | 71 | 8.0 | 91.3 | 14.1 | 26 |
|  |  | Placebo | 161 | 87.6 | 45 | 70 | 7.9 | 90.4 | 13.9 |  |
| PIONEER 6 2019 | Standard-of-care treatment | Oral semaglutide 14 mg | 1591 | 84.7 | 68.1 | 66 | 8.2 | 91.0 | 14.7 | 68 |
|  |  | Placebo | 1592 | 90.1 | 68.6 | 66 | 8.2 | 90.8 | 15.1 |  |
| PIONEER 7 2019 | One or two glucose-lowering drugs (metformin, sulphonylureas, SGLT-2 inhibitors, or thiazolidinediones) | Oral semaglutide up to 14 mg | 253 | 83.4 | 57 | 56.9 | 8.3 | 88.9 | 8.6 | 52 |
|  |  | Sitagliptin 100 mg | 251 | 90.8 | 56 | 57.9 | 8.3 | 88.4 | 9.0 |  |
| PIONEER 8 2019 | Insulin ± metformin | Oral semaglutide 3 mg | 184 | 87.0 | 55.4 | 61 | 8.2 | 85.9 | 15.1 | 52 |
|  |  | Oral semaglutide 7 mg | 182 | 81.3 | 56.6 | 60 | 8.2 | 87.1 | 16.2 |  |
|  |  | Oral semaglutide 14 mg | 181 | 79.6 | 47.0 | 61 | 8.2 | 84.6 | 14.1 |  |
|  |  | Placebo | 184 | 88.0 | 57.1 | 60 | 8.2 | 86.0 | 14.8 |  |
| PIONEER 9 2020 | Diet and exercise | Oral semaglutide 3 mg | 49 | 91.8 | 73 | 58 | 8.1 | 71.4 | 7.4 | 52 |
|  |  | Oral semaglutide 7 mg | 49 | 98.0 | 73 | 60 | 8.3 | 71.3 | 7.4 |  |
|  |  | Oral semaglutide 14 mg | 48 | 93.8 | 83 | 61 | 8.0 | 68.0 | 7.9 |  |
|  |  | Liraglutide 0.9 mg | 49 | 100.0 | 82 | 59 | 8.3 | 70.3 | 8.4 |  |
|  |  | Placebo | 48 | 91.7 | 81 | 59 | 8.3 | 74.7 | 6.7 |  |
| PIONEER 10 2020 | OAD | Oral semaglutide 3 mg | 131 | 94.7 | 76 | 59 | 8.2 | 71.5 | 9.4 | 52 |
|  |  | Oral semaglutide 7 mg | 132 | 93.2 | 68 | 58 | 8.3 | 72.7 | 9.3 |  |
|  |  | Oral semaglutide 14 mg | 130 | 88.5 | 77 | 57 | 8.4 | 72.6 | 9.1 |  |
|  |  | Dulaglutide 0.75 mg | 65 | 93.8 | 78 | 61 | 8.4 | 71.2 | 9.9 |  |

Abbreviations: ER, extended release; HbA_1c_, glycated haemoglobin; OAD, oral anti-diabetic drug; PIONEER, Peptide Innovation for Early Diabetes Treatment; SGLT-2, sodium-glucose cotransporter-2; SUSTAIN, Semaglutide Unabated Sustainability in Treatment of Type 2 Diabetes.
