# Supplementary material for: Efficacy and Safety of Subcutaneous and Oral Semaglutide Administration in Patients With Type 2 Diabetes: A Meta-Analysis
Source: Front Pharmacol. 2021 Oct 6;12:695182. doi: 10.3389/fphar.2021.695182 (PMC8526557; doi:10.3389/fphar.2021.695182)
Supplement: Supplementary file 1 [file DataSheet1.zip › supplementary material/Table S2. Results of efficacy outcomes semaglutide versus other GLP-1 RAs.doc]

Table S2. Results of efficacy outcomes semaglutide versus other GLP-1 RAs

| Outcome | Intervention | Comparator | Trial (n) | WMD/RR (95% CI) | *P* value of WMD/RR (95% CI)^*^ | I^2^ (%) | *P* value^#^ |
| --- | --- | --- | --- | --- | --- | --- | --- |
| Change in HbA_1c_ (%) | Subcutaneous semaglutide 0.5 mg | Dulaglutide 0.75 mg | 1 | -0.40 (-0.55, -0.25) | <0.00001 | NA | NA |
|  | Subcutaneous semaglutide 1 mg | Dulaglutide 1.5 mg, exenatide ER 2 mg,  liraglutide 1.2 mg | 3 | -0.58 (-0.74, -0.41) | <0.00001 | 71 | 0.03 |
|  | Oral semaglutide 7 mg | Dulaglutide 0.75 mg,  liraglutide 0.9 mg | 2 | -0.05 (-0.25, 0.15) | 0.62 | 0 | 0.62 |
|  | Oral semaglutide 14 mg | Dulaglutide 0.75 mg,  liraglutide 0.9 mg,  liraglutide 1.8 mg | 3 | -0.23 (-0.39, -0.07) | 0.004 | 0 | 0.51 |
| Change in body weight (kg) | Subcutaneous semaglutide 0.5 mg | Dulaglutide 0.75 mg | 1 | -2.26 (-3.02, -1.50) | <0.00001 | NA | NA |
|  | Subcutaneous semaglutide 1 mg | Dulaglutide 1.5 mg, exenatide ER 2 mg,  liraglutide 1.2 mg | 3 | -3.72 (-4.16, -3.28) | <0.00001 | 0 | 0.86 |
|  | Oral semaglutide 7 mg | Dulaglutide 0.75 mg,  liraglutide 0.9 mg | 2 | -1.41 (-2.29, -0.53) | 0.002 | 47 | 0.17 |
|  | Oral semaglutide 14 mg | Dulaglutide 0.75 mg,  liraglutide 0.9 mg,  liraglutide 1.8 mg | 3 | -2.03 (-2.91, -1.15) | <0.00001 | 74 | 0.02 |
| HbA_1c_ <7.0% | Subcutaneous semaglutide 0.5 mg | Dulaglutide 0.75 mg | 1 | 1.31 (1.15, 1.50) | <0.0001 | NA | NA |
|  | Subcutaneous semaglutide 1 mg | Dulaglutide 1.5 mg, exenatide ER 2 mg,  liraglutide 1.2 mg | 3 | 1.51 (1.15, 1.98) | 0.003 | 93 | <0.00001 |
|  | Oral semaglutide 7 mg | Dulaglutide 0.75 mg,  liraglutide 0.9 mg | 2 | 1.25 (0.99, 1.56) | 0.06 | 0 | 0.50 |
|  | Oral semaglutide 14 mg | Dulaglutide 0.75 mg,  liraglutide 0.9 mg,  liraglutide 1.8 mg | 3 | 1.31 (1.03, 1.67) | 0.03 | 65 | 0.06 |
| HbA_1c_ ≤6.5% | Subcutaneous semaglutide 0.5 mg | Dulaglutide 0.75 mg | 1 | 1.44 (1.19, 1.75) | 0.0002 | NA | NA |
|  | Subcutaneous semaglutide 1 mg | Dulaglutide 1.5 mg, exenatide ER 2 mg,  liraglutide 1.2 mg | 3 | 1.90 (1.35, 2.66) | 0.0002 | 89 | <0.0001 |
|  | Oral semaglutide 7 mg | Dulaglutide 0.75 mg,  liraglutide 0.9 mg | 2 | 1.28 (0.80, 2.05) | 0.29 | 45 | 0.18 |
|  | Oral semaglutide 14 mg | Dulaglutide 0.75 mg,  liraglutide 0.9 mg,  liraglutide 1.8 mg | 3 | 1.47 (1.17, 1.84) | 0.009 | 25 | 0.26 |
| Weight loss ≥5% | Subcutaneous semaglutide 0.5 mg | Dulaglutide 0.75 mg | 1 | 1.93 (1.51, 2.46) | <0.00001 | NA | NA |
|  | Subcutaneous semaglutide 1 mg | Dulaglutide 1.5 mg, exenatide ER 2 mg,  liraglutide 1.2 mg | 3 | 1.92 (1.16, 3.18) | 0.01 | 92 | <0.00001 |
|  | Oral semaglutide 7 mg | Dulaglutide 0.75 mg,  liraglutide 0.9 mg | 2 | 2.51 (1.06, 5.94) | 0.04 | 0 | 0.94 |
|  | Oral semaglutide 14 mg | Dulaglutide 0.75 mg,  liraglutide 0.9 mg,  liraglutide 1.8 mg | 3 | 3.09 (1.30, 7.31) | 0.01 | 67 | 0.05 |
| Weight loss ≥10% | Subcutaneous semaglutide 0.5 mg | Dulaglutide 0.75 mg | 1 | 4.27 (2.19, 8.34) | <0.0001 | NA | NA |
|  | Subcutaneous semaglutide 1 mg | Dulaglutide 1.5 mg, exenatide ER 2 mg,  liraglutide 1.2 mg | 3 | 4.11 (3.09, 5.48) | <0.00001 | 0 | 0.59 |
|  | Oral semaglutide 7 mg | Dulaglutide 0.75 mg,  liraglutide 0.9 mg | 1 | 9.35 (0.55, 158.19) | 0.12 | NA | NA |
|  | Oral semaglutide 14 mg | Dulaglutide 0.75 mg,  liraglutide 0.9 mg,  liraglutide 1.8 mg | 2 | 2.28 (1.40, 3.73) | 0.001 | 0 | 0.40 |

Abbreviations: CI: confidence interval; ER, extended release; GLP-1 RAs, glucagon-like peptide-1 receptor agonists; HbA_1c_, glycated haemoglobin; NA, not applicable; RR, risk ratio; WMD, weighted mean difference.

*The *P* value of WMD/RR (95% CI) relates to the summary effect in a meta-analysis and is from a Z test (Higgins et al., 2017).

^#^The second *P* value relates to statistical heterogeneity between studies and is from a chi-squared test (Higgins et al., 2017).

References: Higgins JPT, Green S. Cochrane Handbook for Systematic Reviews of Interventions (Version 5.2.0, updated June 2017).
