# Supplementary material for: Efficacy and Safety of Subcutaneous and Oral Semaglutide Administration in Patients With Type 2 Diabetes: A Meta-Analysis
Source: Front Pharmacol. 2021 Oct 6;12:695182. doi: 10.3389/fphar.2021.695182 (PMC8526557; doi:10.3389/fphar.2021.695182)
Supplement: Supplementary file 1 [file DataSheet1.zip › supplementary material/Table S3. Results of achieving HbA1c targets and clinically significant weight loss.doc]

Table S3. Results of achieving HbA_1c_ targets and clinically significant weight loss

| Outcome | Intervention | Comparator | Trial (n) | RR (95% CI) | *P* value of RR (95% CI)^*^ | I^2^ (%) | *P* value^#^ |
| --- | --- | --- | --- | --- | --- | --- | --- |
| HbA_1c_ <7.0% | Subcutaneous semaglutide 0.5 mg | Placebo-controlled | 2 | 3.91 (2.16, 7.11) | <0.00001 | 76 | 0.04 |
|  | Subcutaneous semaglutide 1 mg | Placebo-controlled | 4 | 3.76 (2.60, 5.42) | <0.00001 | 82 | 0.0008 |
|  | Subcutaneous semaglutide 0.5 mg | Active-controlled | 6 | 1.75 (1.47, 2.07) | <0.00001 | 84 | <0.00001 |
|  | Subcutaneous semaglutide 1 mg | Active-controlled | 9 | 1.81 (1.54, 2.13) | <0.00001 | 91 | <0.00001 |
|  | Oral semaglutide 7 mg | Placebo-controlled | 3 | 3.44 (1.86, 6.35) | <0.0001 | 77 | 0.01 |
|  | Oral semaglutide 14 mg | Placebo-controlled | 5 | 3.62 (2.50, 5.25) | <0.00001 | 76 | 0.002 |
|  | Oral semaglutide 7 mg | Active-controlled | 3 | 1.27 (1.10, 1.46) | 0.001 | 0 | 0.78 |
|  | Oral semaglutide 14 mg | Active-controlled | 6 | 1.53 (1.25, 1.86) | <0.0001 | 83 | <0.0001 |
| HbA_1c_ ≤6.5% | Subcutaneous semaglutide 0.5 mg | Placebo-controlled | 2 | 5.46 (3.25, 9.16) | <0.00001 | 34 | 0.22 |
|  | Subcutaneous semaglutide 1 mg | Placebo-controlled | 4 | 6.90 (4.07, 11.69) | <0.00001 | 77 | 0.005 |
|  | Subcutaneous semaglutide 0.5 mg | Active-controlled | 6 | 2.52 (1.83, 3.47) | <0.00001 | 89 | <0.00001 |
|  | Subcutaneous semaglutide 1 mg | Active-controlled | 9 | 2.80 (2.15, 3.65) | <0.00001 | 92 | <0.00001 |
|  | Oral semaglutide 7 mg | Placebo-controlled | 3 | 5.64 (1.80, 17.66) | 0.003 | 75 | 0.02 |
|  | Oral semaglutide 14 mg | Placebo-controlled | 5 | 7.45 (3.75, 14.81) | <0.00001 | 76 | 0.002 |
|  | Oral semaglutide 7 mg | Active-controlled | 3 | 1.44 (1.05, 1.96) | 0.02 | 45 | 0.16 |
|  | Oral semaglutide 14 mg | Active-controlled | 6 | 1.90 (1.50, 2.40) | <0.00001 | 72 | 0.003 |
| Weight loss ≥5% | Subcutaneous semaglutide 0.5 mg | Placebo-controlled | 2 | 4.26 (2.83, 6.42) | <0.00001 | 0 | 0.43 |
|  | Subcutaneous semaglutide 1 mg | Placebo-controlled | 4 | 4.53 (2.08, 9.89) | 0.0001 | 92 | <0.00001 |
|  | Subcutaneous semaglutide 0.5 mg | Active-controlled | 6 | 3.93 (2.42, 6.39) | <0.00001 | 88 | <0.00001 |
|  | Subcutaneous semaglutide 1 mg | Active-controlled | 9 | 3.67 (2.14, 6.28) | <0.00001 | 97 | <0.00001 |
|  | Oral semaglutide 7 mg | Placebo-controlled | 3 | 3.20 (1.75, 5.84) | 0.0002 | 50 | 0.13 |
|  | Oral semaglutide 14 mg | Placebo-controlled | 5 | 4.01 (2.81, 5.71) | <0.00001 | 46 | 0.12 |
|  | Oral semaglutide 7 mg | Active-controlled | 3 | 1.99 (1.54, 2.58) | <0.00001 | 0 | 0.86 |
|  | Oral semaglutide 14 mg | Active-controlled | 6 | 2.10 (1.36, 3.24) | 0.0008 | 89 | <0.00001 |
| Weight loss ≥10% | Subcutaneous semaglutide 0.5 mg | Placebo-controlled | 2 | 3.16 (1.38, 7.28) | 0.007 | 0 | 0.90 |
|  | Subcutaneous semaglutide 1 mg | Placebo-controlled | 4 | 5.43 (2.98, 9.89) | <0.00001 | 43 | 0.15 |
|  | Subcutaneous semaglutide 0.5 mg | Active-controlled | 6 | 4.56 (3.14, 6.62) | <0.00001 | 0 | 0.43 |
|  | Subcutaneous semaglutide 1 mg | Active-controlled | 9 | 5.65 (3.61, 8.84) | <0.00001 | 73 | 0.0003 |
|  | Oral semaglutide 7 mg | Placebo-controlled | 2 | 9.42 (2.87, 30.86) | 0.0002 | 0 | 0.46 |
|  | Oral semaglutide 14 mg | Placebo-controlled | 4 | 8.99 (4.32, 18.69) | <0.00001 | 0 | 0.47 |
|  | Oral semaglutide 7 mg | Active-controlled | 2 | 2.59 (1.56, 4.31) | 0.0002 | 0 | 0.36 |
|  | Oral semaglutide 14 mg | Active-controlled | 5 | 2.27 (1.75, 2.95) | <0.00001 | 0 | 0.71 |

Abbreviations: CI: confidence interval; HbA_1c_, glycated haemoglobin; RR, risk ratio.

*The *P* value of RR (95% CI) relates to the summary effect in a meta-analysis and is from a Z test (Higgins et al., 2017).

^#^The second *P* value relates to statistical heterogeneity between studies and is from a chi-squared test (Higgins et al., 2017).

References: Higgins JPT, Green S. Cochrane Handbook for Systematic Reviews of Interventions (Version 5.2.0, updated June 2017).
