# Supplementary material for: Efficacy and Safety of Subcutaneous and Oral Semaglutide Administration in Patients With Type 2 Diabetes: A Meta-Analysis
Source: Front Pharmacol. 2021 Oct 6;12:695182. doi: 10.3389/fphar.2021.695182 (PMC8526557; doi:10.3389/fphar.2021.695182)
Supplement: Supplementary file 1 [file DataSheet1.zip › supplementary material/Table S4. Results of safety outcomes for subcutaneous semaglutide administration.doc]

Table S4. Results of safety outcomes for subcutaneous semaglutide administration

| Outcome | Comparator | Trial (n) | RR (95% CI) | *P* value of RR (95% CI)^*^ | I^2^ (%) | *P* value^#^ |
| --- | --- | --- | --- | --- | --- | --- |
| Hypoglycemic events | Placebo-controlled | 5 | 1.36 (0.78, 2.39) | 0.28 | 57 | 0.06 |
|  | Active-controlled | 9 | 0.73 (0.56, 0.95) | 0.02 | 0 | 0.51 |
| Acute pancreatitis | Placebo-controlled | 3 | 0.78 (0.35, 1.73) | 0.54 | 0 | 0.65 |
|  | Active-controlled | 4 | 1.28 (0.36, 4.48) | 0.70 | 0 | 0.76 |
| Diabetic retinopathy | Placebo-controlled | 4 | 1.16 (0.57, 2.37) | 0.69 | 52 | 0.10 |
|  | Active-controlled | 8 | 0.97 (0.66, 1.45) | 0.90 | 0 | 0.44 |
| Malignant neoplasms | Placebo-controlled | 5 | 0.95 (0.70, 1.30) | 0.76 | 0 | 0.76 |
|  | Active-controlled | 8 | 1.19 (0.56, 2.50) | 0.65 | 20 | 0.20 |
| Gastrointestinal adverse events | Placebo-controlled | 5 | 2.14 (1.61, 2.85) | <0.00001 | 86 | <0.00001 |
|  | Active-controlled | 9 | 2.28 (1.65, 3.14) | <0.00001 | 96 | <0.00001 |
| Cardiovascular events | Placebo-controlled | 5 | 0.81 (0.66, 0.98) | 0.03 | 2 | 0.39 |
|  | Active-controlled | 6 | 0.73 (0.41, 1.32) | 0.30 | 0 | 0.87 |
| Serious adverse events | Placebo-controlled | 5 | 0.91 (0.83, 0.99) | 0.03 | 0 | 0.85 |
|  | Active-controlled | 9 | 1.09 (0.91, 1.32) | 0.35 | 2 | 0.41 |
| All-cause death | Placebo-controlled | 2 | 1.03 (0.73, 1.46) | 0.85 | 0 | 0.98 |
|  | Active-controlled | 6 | 0.71 (0.31, 1.64) | 0.42 | 0 | 0.77 |
| Cardiovascular death | Placebo-controlled | 2 | 0.97 (0.65, 1.46) | 0.90 | 0 | 0.49 |
|  | Active-controlled | 6 | 1.02 (0.35, 2.95) | 0.98 | 0 | 0.90 |

Abbreviations: CI: confidence interval; RR, risk ratio.

*The *P* value of RR (95% CI) relates to the summary effect in a meta-analysis and is from a Z test (Higgins et al., 2017).

^#^The second *P* value relates to statistical heterogeneity between studies and is from a chi-squared test (Higgins et al., 2017).

References: Higgins JPT, Green S. Cochrane Handbook for Systematic Reviews of Interventions (Version 5.2.0, updated June 2017).
