# Supplementary material for: Efficacy and Safety of Subcutaneous and Oral Semaglutide Administration in Patients With Type 2 Diabetes: A Meta-Analysis
Source: Front Pharmacol. 2021 Oct 6;12:695182. doi: 10.3389/fphar.2021.695182 (PMC8526557; doi:10.3389/fphar.2021.695182)
Supplement: Supplementary file 1 [file DataSheet1.zip › supplementary material/Table S5. Results of safety outcomes for oral semaglutide administration.doc]

Table S5. Results of safety outcomes for oral semaglutide administration

| Outcome | Comparator | Trial (n) | RR (95% CI) | *P* value of RR (95% CI)^*^ | I^2^ (%) | *P* value^#^ |
| --- | --- | --- | --- | --- | --- | --- |
| Hypoglycaemic events | Placebo-controlled | 5 | 1.29 (0.71, 2.32) | 0.40 | 52 | 0.08 |
|  | Active-controlled | 6 | 0.74 (0.50, 1.09) | 0.13 | 14 | 0.32 |
| Acute pancreatitis | Placebo-controlled | 5 | 0.26 (0.04, 1.68) | 0.16 | 0 | 0.73 |
|  | Active-controlled | 5 | 0.78 (0.17, 3.60) | 0.75 | 0 | 0.84 |
| Diabetic retinopathy | Placebo-controlled | 6 | 1.17 (0.90, 1.52) | 0.23 | 0 | 0.80 |
|  | Active-controlled | 6 | 1.43 (0.76, 2.70) | 0.27 | 43 | 0.12 |
| Malignant neoplasms | Placebo-controlled | 6 | 0.80 (0.38, 1.70) | 0.57 | 21 | 0.28 |
|  | Active-controlled | 6 | 1.35 (0.74, 2.48) | 0.32 | 0 | 0.43 |
| Gastrointestinal adverse events | Placebo-controlled | 6 | 3.20 (2.58, 3.97) | <0.00001 | 43 | 0.12 |
|  | Active-controlled | 6 | 1.98 (1.22, 3.19) | 0.005 | 96 | <0.00001 |
| Cardiovascular events | Placebo-controlled | 5 | 0.83 (0.62, 1.12) | 0.22 | 0 | 0.76 |
|  | Active-controlled | 5 | 0.87 (0.51, 1.49) | 0.61 | 0 | 0.80 |
| Serious adverse events | Placebo-controlled | 6 | 0.90 (0.73, 1.13) | 0.37 | 22 | 0.27 |
|  | Active-controlled | 6 | 0.97 (0.76, 1.24) | 0.82 | 16 | 0.31 |
| All-cause death | Placebo-controlled | 6 | 0.56 (0.35, 0.90) | 0.02 | 0 | 0.75 |
|  | Active-controlled | 6 | 0.72 (0.29, 1.76) | 0.47 | 0 | 0.76 |
| Cardiovascular death | Placebo-controlled | 4 | 0.55 (0.31, 0.98) | 0.04 | 0 | 0.85 |
|  | Active-controlled | 6 | 0.60 (0.12, 2.95) | 0.53 | 0 | 0.51 |

Abbreviations: CI: confidence interval; RR, risk ratio.

*The *P* value of RR (95% CI) relates to the summary effect in a meta-analysis and is from a Z test (Higgins et al., 2017).

^#^The second *P* value relates to statistical heterogeneity between studies and is from a chi-squared test (Higgins et al., 2017).

References: Higgins JPT, Green S. Cochrane Handbook for Systematic Reviews of Interventions (Version 5.2.0, updated June 2017).
